# HDAC Inhibition Induces Transient Phenotypic Inertia in Dormant OCCC Spheroids by Derepression of Cell Cycle Genes

**DOI:** 10.3390/cells15080673

**Published:** 2026-04-10

**Authors:** Sylvia Cheng, Bart Kolendowski, Yudith Ramos-Valdes, Trevor G. Shepherd, Gabriel E. DiMattia

**Affiliations:** 1 Mary & John Knight Translational Ovarian Cancer Research Unit, Verspeeten Family Cancer Centre, London, ON N6A 4L6, Canada; sche243@uwo.ca (S.C.); bkolendo@uwo.ca (B.K.); yudithramos@yahoo.es (Y.R.-V.); tshephe6@uwo.ca (T.G.S.); 2 Department of Biochemistry, Western University, London, ON N6A 3K7, Canada; 3 London Health Sciences Centre Research Institute, London, ON N6A 4L6, Canada; 4 Department of Oncology, Western University, London, ON N6A 3K7, Canada; 5 Department of Anatomy & Cell Biology, Western University, London, ON N6A 3K7, Canada; 6 Department of Obstetrics & Gynaecology, Western University, London, ON N6A 3K7, Canada

**Keywords:** clear cell, ovarian, cancer, cell line, spheroid, HDAC inhibitor, dormancy, Entinostat, ACY1215

## Abstract

Multicellular cancer cell aggregates, termed spheroids, are anoikis-resistant, avascular, heterogeneous structures responsible for transcoelomic metastasis of ovarian clear cell carcinoma (OCCC). OCCC is a rare subtype of ovarian cancer with high ARID1A gene mutation rates, resulting in genome-wide changes to H3K27Ac levels and histone deacetylase (HDAC) function. Our study investigated the utility of HDAC inhibitor (HDACi) treatment and H3K27Ac dynamics in OCCC spheroids. By comparing KOC-7c and 105C OCCC cell lines, which have opposing abilities to proliferate as spheroids, we revealed that KOC-7c and 105C spheroids differentially regulated H3K27Ac levels, which correlated with the sensitivity of KOC-7c and the resistance of 105C spheroids to H3K27Ac-altering HDACi treatment. RNA-seq of Entinostat-treated versus vehicle-treated spheroids resulted in a dramatic change in the 105C spheroid transcriptome such that it more closely resembled the proliferative KOC-7c transcriptome over the short term. Comparative pathway analysis identified preferential de-repression of a G2/M checkpoint gene program in 105C spheroids upon Entinostat treatment when compared directly to the KOC-7c spheroids. Our results suggest that the utility of HDACi in OCCC is highly context-dependent.

## 1. Introduction

The contribution of the epigenome to the metastasis of epithelial ovarian cancer (EOC), especially the most prevalent histotype, high-grade serous ovarian cancer (HGSOC), is well established when focused on DNA methylation [1,2,3,4,5]. Post-translational modification of histones (i.e., H3K27Ac and H3K27me3) is increasingly appreciated as a key mechanism of control over the EOC cell phenotype [6,7,8,9,10,11,12]. Metastasis of EOC is partially due to the dissemination of cancer cells from the primary tumor site to form multicellular aggregates, termed spheroids. Spheroids are anoikis-resistant and subsequently attach to other organ surfaces within the peritoneal cavity to form secondary metastatic lesions [13,14,15,16,17]. Our goal was to understand the epigenomic changes that occurred during spheroid formation and how they either lead to spheroid cell dormancy, which provides resistance to DNA-damaging agents, or continued spheroid proliferation [18]. Moreover, we asked if the proliferative potential of spheroid cells warrants the use of specific drugs to disrupt epigenome homeostasis to reduce their viability.

Spheroid formation in EOC is common to all histotypes. We focused on a rare histotype of EOC, ovarian clear cell cancer (OCCC), which comprises 10–25% of EOC depending on geographical location [19,20,21,22]. OCCC is typically treated with debulking surgery followed by the gold-standard chemotherapeutics used for HGSOC, paclitaxel, and platinum-based drugs, which are often ineffective [23,24]. OCCC is distinguished from the other EOC histotypes through its endometriotic cell of origin, which undergoes retrograde transport to the peritoneal cavity during endometriosis and implants on the surface of the ovary [25,26,27]. Many sources convincingly demonstrate that endometriotic tissue cycling in an ectopic location undergoes inflammation and subsequently transforms into OCCC [27,28,29,30,31,32,33,34]. This histotype is also distinct from other EOCs based on its gene mutation profile. Unlike HGSOC, where *TP53* is universally mutated, *TP53* is rarely mutated in OCCC. The mutational profile of OCCC includes frameshift truncating mutations in the SWI/SNF complex member ARID1A, activating mutations in PIK3CA, and loss-of-function mutations in PTEN [35,36,37]. Mutations in the latter two genes result in a significant upregulation of the growth-promoting AKT signaling pathway, which is documented in OCCC cell lines 105C, TOV-21G, KOC-7c, and RMG-V [38].

The loss of ARID1A results in significant epigenomic changes in OCCC cancer cells, including changes in H3K27Ac density due to a loss of interaction between ARID1A and p300 [39,40], activation of HDAC6 [41], changes to direct interaction with other HDACs [42], and HDAC access to specific genomic loci [11,43,44,45,46]. Therefore, the use of HDAC inhibitors (HDACi) may preferentially impact the balance of H3K27Ac marks in ARID1A-mutant cells, which may have a detrimental impact on cell viability. Specifically, we are interested in the contribution of the epigenome to the viability of OCCC spheroids and whether the epigenome presents a therapeutic target. Utilizing a panel of OCCC cell lines, we interrogated changes to the global abundance of histone H3 marks and compared the response of proliferating and dormant OCCC spheroids to HDACi treatment. We hypothesized that OCCC spheroids, which proliferate in suspension culture, would respond differently to epigenome-modifying drugs and that the global abundance of specific H3 marks would change upon spheroid formation. Identifying specific histone marks that change upon spheroid formation will identify the class of epigenome agents that could be used to disrupt spheroid cell viability. Previous studies show that spheroids significantly alter their gene expression profile to remain viable and, in some cases, proliferate in suspension culture [47,48,49,50,51]. Our analyses, using human OCCC cell lines, indicate that H3K27Ac levels change substantially between 2D monolayer culture and spheroid culture. Therefore, we focused on disrupting H3K27Ac levels across the genome using HDACi. Here, we demonstrated that forcing the maintenance of genomic H3K27Ac levels with HDACis (Entinostat and ACY1215) can have dichotomous effects on different OCCC cell lines in spheroid form. We further examined how the spheroid cell transcriptome responds to Entinostat treatment by performing RNA-seq to identify pathways significantly altered by the inhibition of class I HDACs. We found that Entinostat impacted the dormant 105C spheroid cell genome much more dramatically than the highly proliferative KOC-7C spheroids. Interestingly, disrupting the balance of H3K27Ac globally resulted in a significant elevation in the expression of genes involved in the regulation of G2/M checkpoint of the cell cycle, uniquely in the 105C spheroid. The KOC-7C spheroid cell transcriptome also showed distinctive changes in gene expression upon HDACi treatment, but specific pathway analysis did not reveal mechanisms that were associated with cell cycle control.

## 2. Materials and Methods

### 2.1. Cell Culture and Reagents

We have previously described the OCCC cell lines used in this study and their origin [38,52]. The maintenance of the OCCC cell lines for this work is as described previously [38,52]. In brief, the culture medium for all cell lines was Dulbecco’s Modified Eagle Medium/Ham’s F12 (Invitrogen, Waltham, MA, USA, catalogue #11330057) with 10% FBS (Wisent, catalogue #098-150-CL). All cell lines were propagated as monolayer cultures grown at 37 °C with 5% CO_2_. Passaging of cells occurred at 80–90% cell confluency, where media were aspirated, and the monolayer was washed with Phosphate-Buffered Saline (PBS) (Wisent, Saint-Jean-Baptiste, QC, Canada, catalogue #311-425-CL). Cells were detached using trypsin/EDTA, and cells were reseeded at a 1:5–1:10 dilution to a new culture vessel. Autonomous OCCC cell line spheroid formation was performed using detached cells seeded to ultralow attachment (ULA) plates (Corning, Corning, NY, USA, catalogue #3471 (6-well), 3473 (24-well), and 3474 (96-well)). Authentication of cell lines was done using short tandem repeat (STR) analysis at the Centre for Applied Genomics (TCAG) in Toronto, Ontario, Canada, and we cross-referenced the data with the compendium of STR fingerprints housed in the CLASTR 1.4.4 database (https://web.expasy.org/cellosaurus-str-search/, accessed on 8 March 2021).

HDAC inhibitors—Entinostat (#HY-12163), ACY-1215 (#HY-16026), Panobinostat (#HY-10224), and Belinostat (#HY-10225)—were purchased from MedChemExpress (Monmouth Junction, NJ, USA). AlamarBlue™ cell viability reagent (#DAL1100) and Trypan Blue Solution (#15250061) were purchased from ThermoFisher Scientific (Waltham, MA, USA). Hank’s balanced salt solution (HBSS; #311-515-CL) was purchased from Wisent (Saint-Jean-Baptiste, QC, Canada). Protease inhibitors for use in protein lysates for Western blot were purchased from MilliporeSigma (Burlington, MA, USA, SIGMAFAST Protease inhibitor tablets; #S8820-20TAB)

For Western blot experiments, the primary and secondary antibodies used in this study are listed in Table 1 (below). The commercial supplier of each antibody is also supplied.

### 2.2. Nuclear Histone Lysate Preparation for Immunoblotting

We used the following protocol to avoid including non-chromatin-integrated cytoplasmic histones in our analyses. To prepare nuclear histone extracts, adherent cells were washed and scraped in PBS and transferred to a microcentrifuge tube. Spheroid cells were collected from ULA culture plates into conical tubes and centrifuged at 2400× *g* for 5 min and then washed with PBS. Cells were pelleted by centrifugation for 10 s at 10,000× *g*; then, the supernatant was removed and washed twice with the nonionic, non-denaturing detergent 0.1% *v*/*v* IGEPAL CA-630 in PBS to lyse plasma membranes but not nuclear membranes. The supernatant was removed, and pellets were suspended in 0.2N HCl (4 × 10^7^ nuclei/mL) and incubated without agitation at 4 °C overnight to extract the histone from the chromatin. After the overnight incubation, cell debris was pelleted by centrifugation, and the supernatant containing the histones was transferred to a new microcentrifuge tube. The acid in the extract was neutralized with 2M NaOH (volume of 1:10 supernatant); then, a 10× protease inhibitor cocktail was added to prevent histone protein degradation.

### 2.3. Immunoblotting

Whole-cell lysates were generated as previously described [38]. Protein lysates (20–30 µg for whole cells or 1 µg for acid-extracted histone lysate) were loaded onto Bolt 4–12% Bis-Tris Plus polyacrylamide gels (Invitrogen, Waltham, MA, USA, catalogue #NW04122BOX), and the proteins were electrophoretically separated according to the manufacturer’s instructions. Separated proteins were then transferred to PVDF (polyvinylidene difluoride) membranes using the Bio-Rad “Trans-Blot Turbo” system. The blocking and washing of the PVDF membranes were conducted in Tris-buffered saline with Tween 20 (TBST; composed of 20 mM Tris, 150 mM NaCl, and 0.1% Tween 20). Membrane blocking was done with either 5% skimmed milk or 5% bovine serum albumin (BSA) in TBST depending on the requirements of the primary antibodies used.

Primary antibodies were diluted according to the manufacturer’s recommendations, incubated with the membranes overnight at 4 °C, and then sequentially washed three times for 10 min each with TBST. Subsequently, membranes were reacted with either mouse or rabbit secondary antibodies for one hour at room temperature. Afterward, the membranes underwent washing three times for 10 min each with TBST. Enhanced chemiluminescence (ECL) detection of the protein of interest was achieved by incubating membranes in Immobilon Forte Western HRP Substrate (Millipore, Burlington, MA, USA, #WBLUF0500) for 2 min. The chemiluminescent images emitted from the transfer membrane were acquired using the Bio-Rad ChemiDoc imaging system, which also provided signal intensity quantification.

Protein transfer membranes were stripped of primary and secondary antibodies to probe with an antibody targeted against the protein loading control. Membranes were incubated with a harsh stripping buffer (62.5 mM Tris-HCl, pH 6.8, 2% SDS, 100 mM Beta-mercaptoethanol) for 30 min in a 50 °C rocking water bath. Membranes subsequently underwent 2 × 5 min TBST washes and were blocked with either 5% *m*/*v* BSA or milk in TBST for 30 min, after which the membranes were incubated in a primary antibody (i.e., total H3 for specific histone marks) diluted in the appropriate buffer overnight at 4 °C. Subsequent steps were performed as described above.

### 2.4. Time-Course Experiments Assessing Global Levels of H3K27Ac in OCCC Cell Lines

For monolayer cultured time course experiments, OCCC cell lines were seeded in 6-well adherent culture plates at densities that yielded a 90% confluent plate at the time of cell harvest and protein lysate preparation. Monolayer cells were treated with different HDAC inhibitors 24 h after seeding. For spheroids, cells were seeded at 5 × 10^5^ cells/well in 6-well ULA culture plates and either treated at the time of seeding or maintained untreated to provide control lysates. Nuclear histone protein was acid-extracted according to the above procedure at every treatment time point and used for Western blotting.

### 2.5. Half-Maximal Inhibitory Concentration Determination

Adherent cells were seeded at 1.5–3 × 10^3^ cells/well in 96-well adherent plates (Sarstedt, Nümbrecht, Germany, #83.3924), and spheroid cells were seeded at 2–5 × 10^5^ cells/well in 96-well flat-bottom ULA culture plates (Corning, NY, USA). Cells were treated with the listed drug concentrations after overnight attachment for adherent cells and at the time of seeding for spheroid cells in triplicate. After 3 days of treatment, alamarBlue™ cell viability reagent was used to quantify treatment efficacy. Absorbance at 590 nm was taken using the Agilent BioTek Synergy H1 plate reader (Mississauga, ON, Canada) after 3–24 h incubation. Absorbance measurements for the lowest drug concentration were used to establish a baseline of 100% viability; then, viability measurements for each concentration were taken as a percentage of the baseline. GraphPad Prism was used to calculate IC50 concentrations by fitting a curve to the baseline-corrected data using non-linear regression (equation: log(inhibitor) vs. response—variable slope (four parameters)).

### 2.6. Clonogenic Assays

Cells were seeded at 150–300 cells/well in 24-well adherent culture plates (Sarstedt, #83.3922). Cells were allowed to attach to culture plates overnight and then treated with the listed concentrations for 7 days. Media and drugs were not changed or supplemented for the duration of the treatment period; then, the alamarBlue™ cell viability reagent was used to determine the relative viability of cells at each concentration. The IC50 for colony formation was calculated using the method described above. Subsequently, plates were stained with HEMA 3 Stat Pack (ThermoFisher Scientific, #23-123869) according to the manufacturer’s instructions to visualize colonies.

### 2.7. RNA-Seq Preparation and Analysis

In brief, 105C and KOC-7c cells were treated with either DMSO or Entinostat (1 µM) and cultured for 3 days on ULA plates. Total RNA was extracted with the RNeasy Kit (Qiagen, Venlo, The Netherlands, #74106), quantified, and assessed for integrity using 5K/RNA/Charge Variant Assay LabChip and an RNA Assay Reagent Kit (Perkin Elmer, Waltham, MA, USA). Sequencing libraries were prepared using 250 ng of total RNA. Briefly, mRNA enrichment and library construction were performed using the Illumina Stranded mRNA Prep Kit (Illumina, San Diego, CA, USA), according to the manufacturer’s recommendations. Libraries were quantified using the KAPA Library Quantification Kits—Complete kit (Universal) (Kapa Biosystems, Wilmington, MA, USA), and the average fragment size was determined using Fragment Analyzer 5300 (Agilent, Santa Clara, CA, USA). Libraries were normalized, pooled, and denatured in 0.02N NaOH and neutralized with a pre-load buffer. The final pool was loaded at 150 pM on Illumina NovaSeq X Plus 25B flow cell according to the manufacturer’s recommendations. Sequencing was performed in the paired-end mode for 2 × 100 cycles. A PhiX library was included as a 1% spike-in control. Demultiplexing and FASTQ generation were performed using BCL Convert 4.2.4. Initial sequencing data were carried out on the Galaxy platform. Raw reads were evaluated using FastQC (v. 0.74), aligned to the human genome (hg38) using STAR (v2.7.11b) with default settings, and quantified using featureCounts (v2.1.1). Differential expression analysis was performed using limma-voom (v3.58.1) following TMM normalization. Lowly expressed genes were excluded using a cutoff of CPM ≤ 0.5 in fewer than 2 samples.

Gene set enrichment analysis (GSEA) was performed using GSEA software (v4.4.0; Broad Institute, MIT and Harvard), as described by Subramanian et al. 2005 [53]. Genes were ranked by multiplying the sign of the log-transformed fold change by −log10 (*p*-value) derived from the limma output. Principal component analysis (PCA) was assessed using the sklearn module from the Python library (v3.11) and visualized using standard Python libraries. Unless otherwise indicated, all downstream analyses and figures, including but not limited to volcano plots, heatmaps, pair-wise comparisons, and bar graphs, were generated from raw data or tool outputs (e.g., limma-voom’s “normalized counts” output) using custom Python scripts with standard data science modules.

### 2.8. Spheroid Reattachment Assay

OCCC cell lines were seeded in 24-well ULA culture plates at 2.5 × 10^4^ cells/well for KOC-7c and 1 × 10^5^ cells/well for 105C. Cells were treated at the time of seeding for HDACi treatments and 3 days post-seeding for HDACi treatments in biological triplicates. After 3 days of treatment, spheroid cells were transferred (along with media) well by well into 24-well adherent culture plates and then supplemented with additional complete media. For KOC-7c cells, spheroid cells were allowed to attach to cell culture substrata overnight, while 48 h was allowed for 105C spheroids. Once attached, cell viability was quantified using alamarBlue cell viability reagent, and fluorescence was measured at 590 nm using the Agilent BioTek Synergy H1 plate reader.

### 2.9. Trypan Blue Exclusion Cell Counting of Spheroids

All experiments were conducted in 24-well ULA plates. For long-term spheroid proliferation experiments, cells were seeded at 1 × 10^5^ cells/well and treated at the time of seeding in triplicate. For short-term cell counting, KOC-7c and TOV-21G cells were seeded at 2.5 × 10^4^ cells/well, and 105C cells were seeded at 1 × 10^5^ cells/well. At indicated time points, spheroids from each well were collected in a microcentrifuge tube, pelleted at 5000× *g*, and then washed with PBS. After pelleting again, as much PBS was removed as possible; then, spheroids were incubated in 50–300 μL 0.25% trypsin/EDTA in a 37 °C water bath for 30 min to separate spheroids into single cells. Trypsin was neutralized with an equivalent volume of FBS. Trypan blue dye was added at a volume equivalent to the total volume of trypsin + FBS; then, cell counting was conducted using the Bio-Rad TC20 Automated Cell Counter (Hercules, San Mateo, CA, USA). For long-term experiments, fresh media not containing the drug were supplemented on day 5.

### 2.10. Spheroid Immunostaining

ES2 cells were seeded at 5 × 10^5^ cells/well, and 105C cells were seeded at 1 × 10^6^ cells/well in 6-well ULA plates and treated with either 1 μM Entinostat or Vehicle (DMSO) diluted in complete media. Sample preparation and immunostaining procedure were performed as detailed in Borrelli et al. 2025 [54].

### 2.11. Statistics

Statistical analyses were performed using GraphPad Prism v10.4.1. Statistical tests performed include one-way or two-way ANOVA followed by Tukey’s multiple-comparison test or Dunnett’s multiple-comparison test, paired and unpaired two-sample *t*-tests, and one-sample *t*-tests. Specific analysis details are described in the figure legends.

## 3. Results

### 3.1. Genomic H3K27Ac Levels Are Detectable and Dynamic in OCCC Cell Line Spheroid Cells

**Figure 1:**
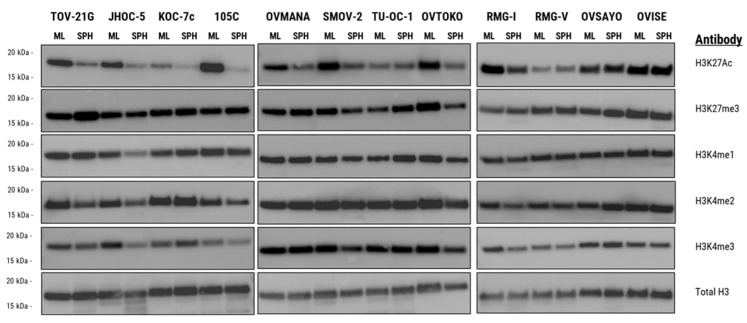
Immunoblot survey of global histone-modification levels in 12 ovarian clear-cell carcinoma (OCCC) cell lines cultured for 3 days either as adherent monolayers (MLs) or as spheroids in ultra-low attachment plates (SPH). Nuclei were isolated, histones acid-extracted, and 1 µg of total histone per lane was resolved by SDS-PAGE. Membranes were probed with the indicated anti-histone modification antibodies, and total H3 served as a loading control. TOV-21G, JHOC-5, 105C, OVMANA, SMOV-2, OVTOKO, and RMG-I cell lines displayed an obvious decrease in the H3K27Ac mark in spheroids relative to their proliferating monolayer cells. In contrast, the other H3 marks assayed showed a remarkably consistent global level regardless of culture condition.

OCCC cells cultured in ULA autonomously aggregate into 3D structures with unique biology that recapitulates patient spheroid biology better than traditional 2D monolayer culture [17,54,55,56,57,58]. We surveyed the global level of the key H3K27 marks to determine which, if any, showed a detectable change in Western blot signal intensity between monolayer and spheroid cells. The objective was to identify an epigenetic mark where abundance was linked to 3D spheroid formation. Interestingly, H3K27me3 levels did not change significantly between monolayers and spheroids among the 12 cell lines tested (Figure 1). However, there was a large decrease in H3K27Ac levels in several OCCC cell line spheroids compared to their monolayer counterparts, namely, TOV-21G, JHOC-5, 105C, OVMANA, SMOV-2, OVTOKO, and RMG-I (Figure 1).

**Figure 2:**
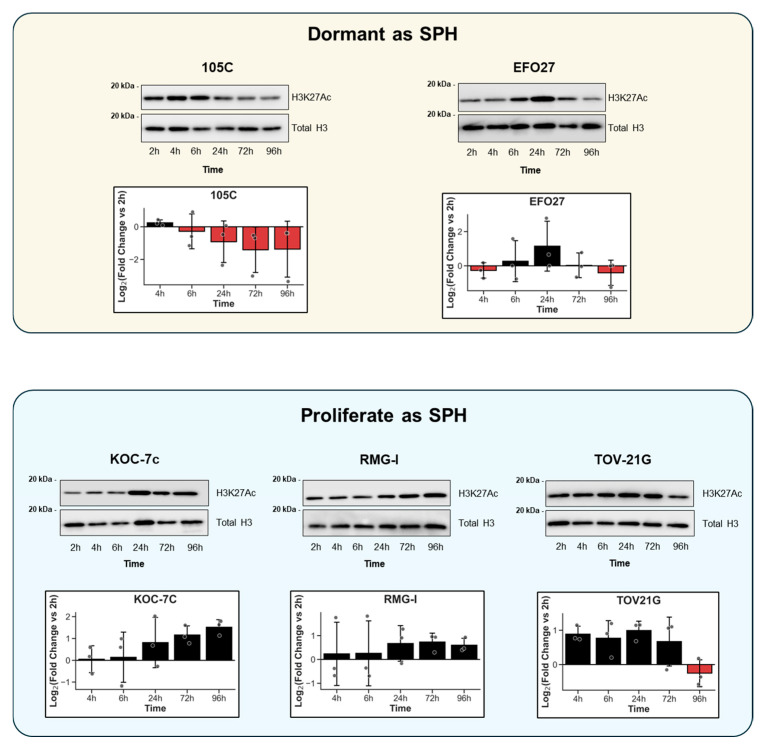
Acetylation at K27 of Histone 3 (H3K27Ac) is detectable and varies in human OCCC cell line spheroids over time. OCCC cells were seeded at a density of 500 K cells/well in 6-well ULA plates. Time points represent time elapsed after seeding to spheroid culture. Acid-extracted nuclear histone protein was isolated at each time point and used for Western blot detection of global H3K27Ac and total histone 3 (H3) protein levels. H3K27Ac signal was normalized to its respective total H3 signal. Normalized values were used to calculate Log2fold-change (time point/2 h) for each time point. Graphical representations of H3K27Ac levels at different time points over the 96 h assay period show that H3K27Ac levels can vary significantly over time. Note that the Y-axis provides values relative to the H3K27Ac signal at the 2 h timepoint.

We also investigated if and how H3K27Ac levels changed over a span of 96 h in several OCCC cell line spheroids, especially during stages of early spheroid formation (2–24 h). Using three cell lines that proliferate as spheroids (KOC-7c, TOV-21G, and RMG-I) and two cell lines that do not proliferate (105C, EFO-27) [38,52], acid-extracted nuclear histone proteins were collected at specified time points post-seeding into ULA culture (Figure 2). KOC-7c, TOV-21G, and RMG-I spheroids appeared as a collection of loose cell clusters, while 105C and EFO-27 spheroids formed distinct compact cellular aggregates. H3K27Ac levels were detectable by Western blot in all OCCC cell line spheroids over the entire time course. Interestingly, a slight reduction in H3K27Ac levels was seen in 105C and EFO27 cell line spheroids after the 24 h timepoint of spheroid formation for cell lines that proliferate in spheroid form (KOC-7c, RMG-I, and TOV-21G). The global level of H3K27Ac generally increased over time, although the TOV-21G cell line displayed a reproducible decline in H3K27Ac levels at the 4-day timepoint.

Overall, these results show that changes in the steady-state global levels of H3K27Ac can occur in a cell line-specific way as OCCC cells acclimate to the loss of cellular substratum as spheroids. Importantly, this data shows that H3K27Ac levels are detectable via Western blot and implies that regulating H3K27Ac levels may be critical for certain aspects of spheroid biology and viability regardless of the spheroid cell proliferative state or potential. Therefore, we focused our studies on the disruption of H3K27Ac homeostasis in OCCC cell line spheroids with the use of HDAC inhibitors (HDACi). HDACi treatment may impact the epigenomic environment of OCCC cells, resulting in changes in the transcriptome that could lead to cell death. This led to the hypothesis that disrupting downregulation and maintaining a high level of global H3K27Ac in spheroid cells would significantly and negatively impact their viability, thereby representing a therapeutic advantage.

### 3.2. HDACi Treatment Elevates Global H3K27Ac Levels in OCCC Cell Lines

**Figure 3:**
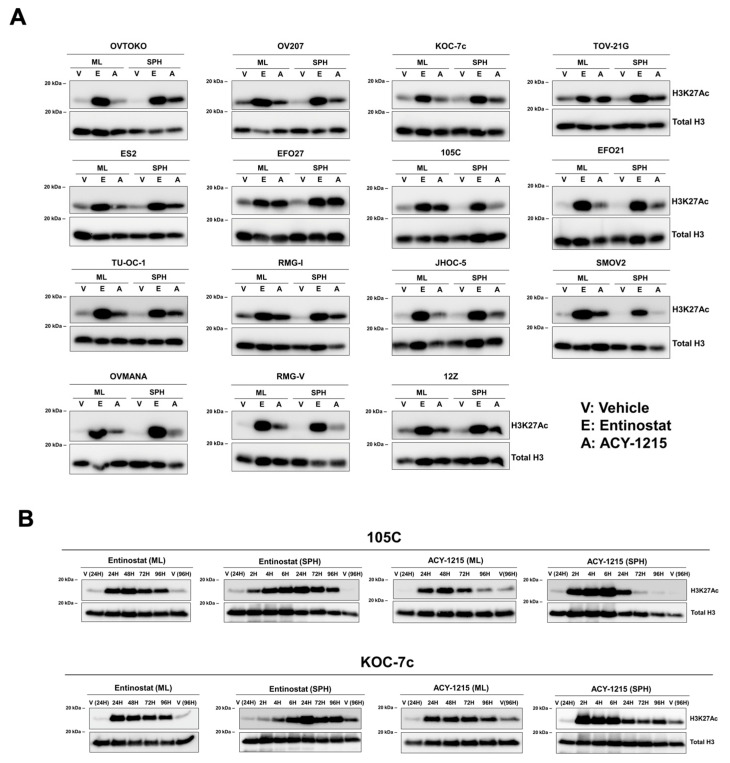
OCCC cell lines are universally sensitive to the effects of two different HDAC inhibitors under different culture conditions. (**A**) Screen of 15 OCCC cell lines for HDACi response. OCCC cell lines were seeded in adherent culture (monolayer; ML) at 500 K cells/10 cm dish and ULA culture for forming spheroids (SPHs) at 500 K cells/well in 6-well ULA and treated with 1 µM Entinostat or 5 µM ACY-1215 after cell attachment for ML and at the time of seeding for SPH. On day 3 of treatment, acid-extracted nuclear lysates were collected for Western blotting analysis of H3K27Ac and total H3. Both 3D spheroids and monolayer cells were highly sensitive to Entinostat and ACY-1215, as indicated by the strong elevation in H3K27Ac levels. Only SMOV2 spheroids appeared refractory to ACY-1215, as it did not elicit a noticeable accumulation of H3K27Ac. (**B**) Assessing the durability of the HDACi response on H3K27Ac levels over 96 h. KOC-7c and 105C cell lines were seeded in 6-well adherent or ULA spheroid culture at 100 K and 500 K cells/well, respectively. Cells were treated with 1 µM Entinostat or 5 µM ACY-1215. Monolayer cells were treated after attachment, while spheroid cells were treated at the time of seeding. Time points represent time elapsed after dosing. Nuclear histone protein was collected at each time point and used for Western blotting. The kinetics of H3K27Ac accumulation upon HDACi treatment were similar between the two cell lines for both drugs. Elevation of H3K27Ac was faster under ACY-1215 treatment but was more durable under Entinostat treatment.

Inhibition of HDAC proteins would allow genomic histone acetylation marks to accumulate as histone acetylase (HAT) proteins continue their enzymatic activity. Hence, an elevation in global acetylation levels was used as the readout for HDAC inhibition. The literature suggests that 1 µM Entinostat (ENT) [59,60,61,62,63] and 5 µM ACY-1215 (ACY) [64,65,66,67,68] were effective starting concentrations; hence, we assessed global H3K27Ac levels in OCCC cells upon treatment at these concentrations for 3 days in 15 OCCC cell lines in both monolayer and spheroid culture conditions, with the objective of determining if all OCCC cell lines were equally sensitive to HDACi and whether some lines represent unique resistant cell line models. Both ENT and ACY treatment elevated H3K27Ac levels compared to vehicle treatment in both culture conditions in all cell lines tested, except for SMOV2 in the spheroid culture, where ACY treatment did not elicit a noticeable accumulation of H3K27Ac (Figure 3A). Overall, ENT treatment elicited a more robust increase in H3K27Ac than ACY in almost all cell lines and culture conditions.

Next, we assessed the kinetics of the accumulation and potential loss of H3K27Ac over 96 h after a single dose of HDACi in KOC-7c and 105C monolayer and spheroid cells (Figure 3B). Cells were treated with 1 µM ENT and 5 µM ACY at the time of seeding for spheroids and after attachment for monolayer cells. The kinetics of H3K27Ac accumulation under HDACi treatment were similar between the two cell lines for both drugs. Elevation of H3K27Ac was faster under ACY treatment but was more durable under ENT treatment. H3K27Ac levels were clearly elevated after just 2 h of ENT treatment, with levels peaking at 24 h, and H3K27Ac levels were still elevated after 96 h of treatment in both culture conditions (Figure 3B). ACY treatment resulted in faster accumulation of H3K27Ac, with levels peaking between 2 and 6 h of treatment in spheroids; however, H3K27Ac levels returned to the baseline by 72 h of treatment in spheroid cells and by 96 h in monolayer cells (Figure 3B). This is consistent with the results of Figure 3A, where ACY elicited a weaker H3K27Ac signal 3 days post-treatment in all cell lines. Taken together, a single dose of ENT or ACY increased global histone acetylation in OCCC cells, and the durability and kinetics of drug response were mostly drug-dependent rather than cell-line-dependent.

### 3.3. HDACi Treatment Reduces OCCC Cell Viability in a Dose-Dependent Manner

**Figure 4:**
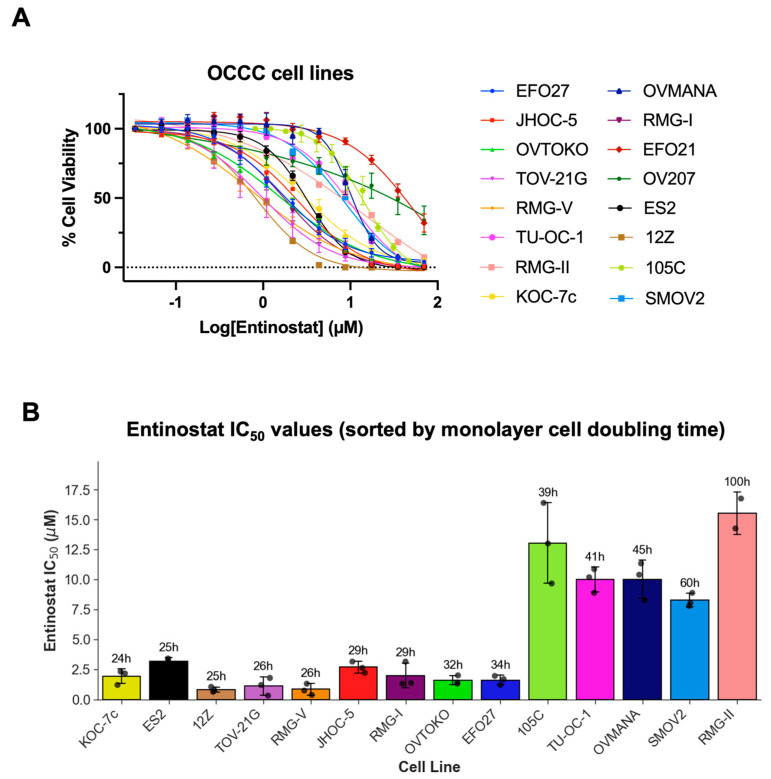
Half-maximal inhibitory concentrations (IC50) of HDACi activity in OCCC cell lines. (**A**) Entinostat IC50 curves for 16 OCCC cell lines in monolayer culture. Cells were seeded in 96-well adherent plates at 1.5–3 K cells/well and treated with increasing concentrations of Entinostat after overnight attachment in triplicate. After 3 days of treatment, alamarBlue™ cell viability reagent was used to quantify treatment efficacy, where viability measurements for the lowest drug concentration were used to establish a baseline of 100% viability, and viability measurements for each concentration were taken as a percentage of the baseline. (**B**) Cell lines are ordered on the x-axis by increasing monolayer culture doubling time. EFO21 and OV207 displayed resistance to the drug at the concentrations tested and did not produce an IC50 value and, therefore, are not included in the graph. Overall, cell lines with the shorter doubling time exhibited greater sensitivity to Entinostat. The actual EC50 values are presented in Supplementary Table S1.

To assess the functional consequences of H3K27Ac elevation after HDACi treatment, the half-maximal growth inhibitory concentration (IC50) for ENT was determined for 16 OCCC cell lines in a monolayer. OCCC cells were treated with increasing doses of ENT for 3 days; then, cell viability was determined using alamarBlue™. ENT induced a dose-dependent decrease in cell viability in all cell lines tested (Figure 4A). ENT IC50s ranged from ~0.8 µM to 50 µM after 3 days of treatment. There was also an association between cell proliferation and ENT IC50; cell lines with faster doubling times tended to have lower IC50 values (Figure 4B). OV207 and EFO21 cell lines were particularly resistant to ENT treatment given that 70 µM was insufficient at fully reducing relative cell viability, indicated by the lack of a bottom plateau in the dose–response curve (Figure 4A). Consistently, OV207 and EFO21 had the third and fourth longest doubling times of the 16 cell lines at 59.7 and 52.2 h, respectively. The fastest doubling time was 24 h (KOC-7c), equating to three cell doublings over the treatment period, while OV207 and EFO21 underwent roughly 1.4 doublings. Given that epigenetic changes are passed onto daughter cells, more cell doublings over the treatment period likely mean that histone modifications brought about by HDACi treatment are propagated to cells of the more proliferative cell lines faster, and changes in cell viability are seen earlier. Therefore, we assessed the viability of these two cell lines after 7 days of ENT treatment, equating to ~3 doubling times. Seven-day treatment with the same doses of ENT elicited 100% cell killing in both cell lines, and the calculated IC50 was 0.717 and 6.22 µM for OV207 and EFO21, respectively (Figure S1).

**Figure 5:**
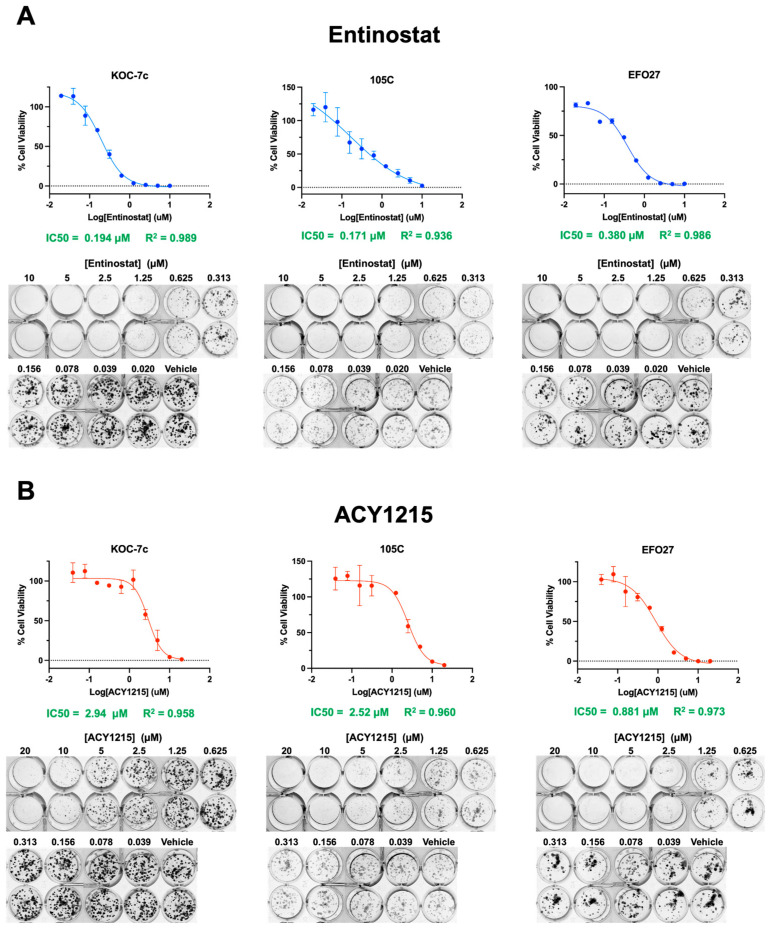
HDACi impedes OCCC cell line colony formation (**A**,**B**). KOC-7c, 105C, and EFO-27 cell lines were seeded at 150–300 cells/well in 24-well adherent culture plates. Cells were treated after attachment and allowed to form colonies over 7 days. AlamarBlue™ was used to calculate IC50 concentrations. Representative images of HEMA 3-stained adherent plates after treatment are shown under the dose–response curves. All three cell lines were more sensitive to HDACi in this assay relative to using a large cell population, given that their IC50s as bulk cells ranged from ~2.5 to 13 µM for ENT and ~5 to 14 µM for ACY (Figure 4). Together, monolayer OCCC cells are sensitive to HDACi treatment through a decrease in cell viability both as bulk and single cells, and cell lines are differentially sensitive to HDACi loosely based on their proliferation rate.

To further assess the viability of OCCC cells under HDACi treatment, we performed clonogenic assays. This is often considered the gold standard for determining the functional consequences of drug response, as viable cells are asked to sustain proliferation in the presence of a treatment [69,70,71]. Unlike assessing bulk cell viability, clonogenic assays can identify the tumorigenic potential of single cells after treatment and may reveal specific subpopulations of cells that confer treatment resistance. Using this assay, cells lack cell–cell contact and support, making this a more robust assessment of drug sensitivity. KOC-7c, 105C, and EFO27 OCCC cell lines were seeded at low densities and treated with ENT or ACY for 7 days. The total cell viability of each well was determined using alamarBlue^TM^; then, the half-maximal inhibitory concentration for colony formation was determined (Figure 5). The IC50 of both ENT and ACY for colony formation between the three cell lines ranged from ~0.2 to 0.4 µM and ~1.0 to 3.0 µM, respectively. All three cell lines were more sensitive to HDACi as single cells than as bulk cells, given that their IC50s as bulk cells ranged from ~2.5 to 13 µM for ENT and ~5 to 14 µM for ACY (Figure 5). Together, monolayer OCCC cells are sensitive to HDACi treatment through a decrease in cell viability both as bulk and single cells, and cell lines are differentially sensitive to HDACi, as correlated with their proliferation rate.

### 3.4. OCCC Spheroids Respond to HDACi Treatment Based on Their Proliferative Abilities

**Figure 6:**
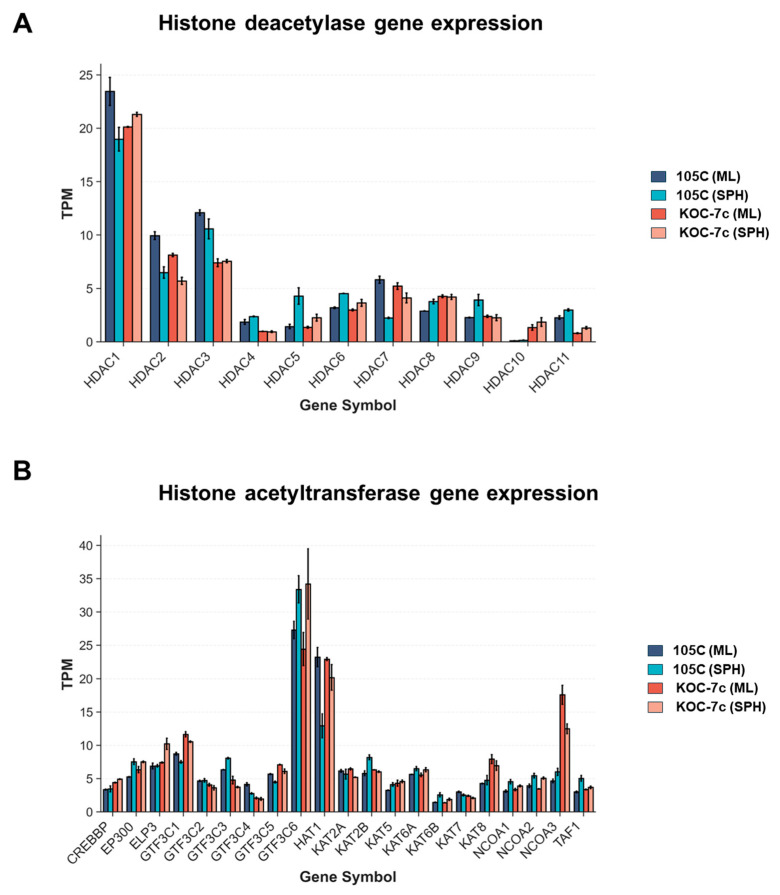
HDAC expression in KOC-7c and 105C monolayer (ML) and spheroid (SPH) cells based on RNA-seq data. Cells were grown as a monolayer or in suspension to form spheroids for 3 days; then, RNA was collected and analyzed by RNA-seq. (**A**) Zn2+-dependent HDACs (**B**) Canonical and putative HATs are shown. Histone deacetylases and histone acetyltransferases are detectable across both cell lines and conditions, with no significant differences between monolayer cells and spheroids.

Given that fast- and slow-growing cells are differentially sensitive to HDACi treatment, we chose two OCCC cell lines, KOC-7c and 105C, for spheroid experiments with a goal of determining if HDACs represent a potential therapeutic target for metastatic OCCC. KOC-7c and 105C share the classic OCCC mutational profile of deactivated ARID1A and PTEN and activated PIK3CA, while demonstrating dramatic differences in proliferation and viability in spheroid form; KOC-7c spheroids are highly proliferative and metabolically active, while 105C spheroids are dormant. First, we assessed transcript levels of HDAC and HAT genes in KOC-7c and 105C monolayer and spheroid cells using RNA-seq from our previous work [38] to determine if HDAC/HAT gene expression may predict or correlate with HDACi sensitivity. HDACs 1, 2, and 3 were the most abundantly expressed HDACs (Figure 6A), and GTF3C6 (General Transcription Factor IIIC Subunit 6) and HAT1 were the most highly expressed HATs (Figure 6B) regardless of culture condition.

**Figure 7:**
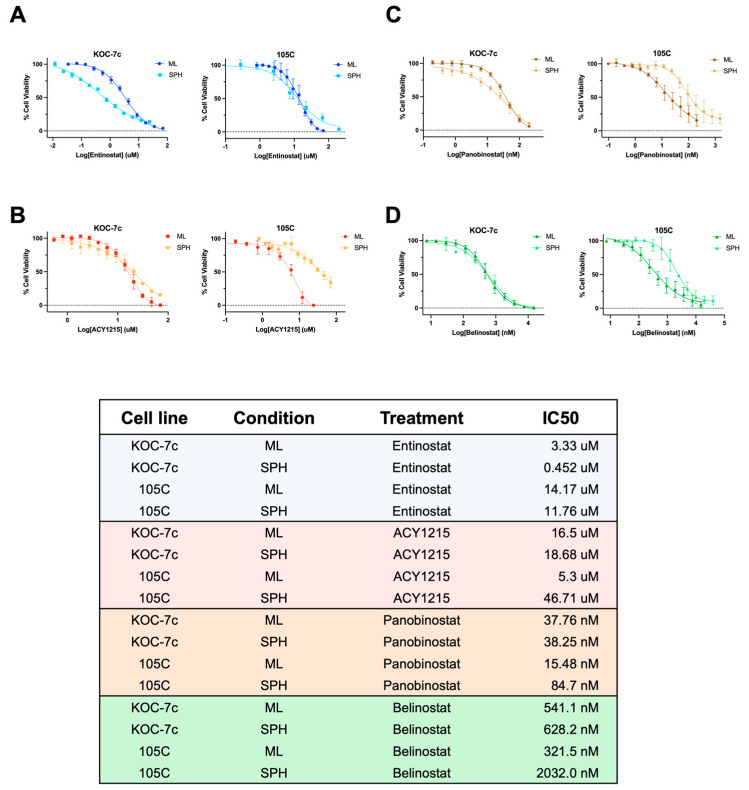
Sensitivity of dormant and proliferative spheroids to HDACi. KOC-7c and 105C OCCC cell lines were seeded in 96-well adherent (monolayer; ML) plates at 1.5–2 K cells/well or in 96-well ULA culture at 20–50 K cells/well to form spheroids (SPHs). Cells were treated with HDACis Entinostat ((**A**); blue), ACY-1215 ((**B**); red), Panobinostat ((**C**); brown), or Belinostat ((**D**); green) after overnight attachment for monolayer cells and at the time of seeding for spheroid cells. After 3 days, alamarBlue™ cell viability reagent was used to quantify treatment efficacy. GraphPad Prism was used to calculate IC50 concentrations.

To ensure that our results were not unique to ENT or ACY1215, we employed the pan-HDAC inhibitors Panobinostat and Belinostat. Moreover, the inclusion of these latter HDAC inhibitors served to determine whether an HDAC inhibitor that targeted all HDACs might be more efficacious than those with a more limited target specificity, like ACY1215. The IC50s of the four HDACi used, Entinostat, ACY-1215, Panobinostat, and Belinostat, were determined for both cell lines in monolayer and spheroid culture (Figure 7). The IC50 for ENT was lower than that of the ACY for both KOC-7c and 105C cell lines in both culture conditions. Overall, proliferating KOC-7c spheroids were more sensitive to ENT treatment than monolayer cells by a factor of 7, while monolayer and spheroid cells were similarly sensitive to ACY treatment. 105C monolayer and spheroid cells were similarly sensitive to ENT, but 105C spheroids were more resistant to ACY treatment than monolayer cells by a factor of 9 (Figure 7A,B). Upon treatment with pan-HDACi, Panobinostat, and Belinostat, 105C spheroid cells, which are dormant, were more resistant to treatment than monolayer cells; 105C spheroids had a ~5.5 to 6-fold higher IC50 concentration for these two inhibitors compared to monolayer cells (Figure 7C,D). KOC-7c monolayer and spheroid cells were similarly sensitive to both inhibitors. In support of our previous results, the dormant spheroid phenotype confers resistance to HDACi treatment, while actively proliferating cells are susceptible to the negative effects of HDAC inhibition.

**Figure 8:**
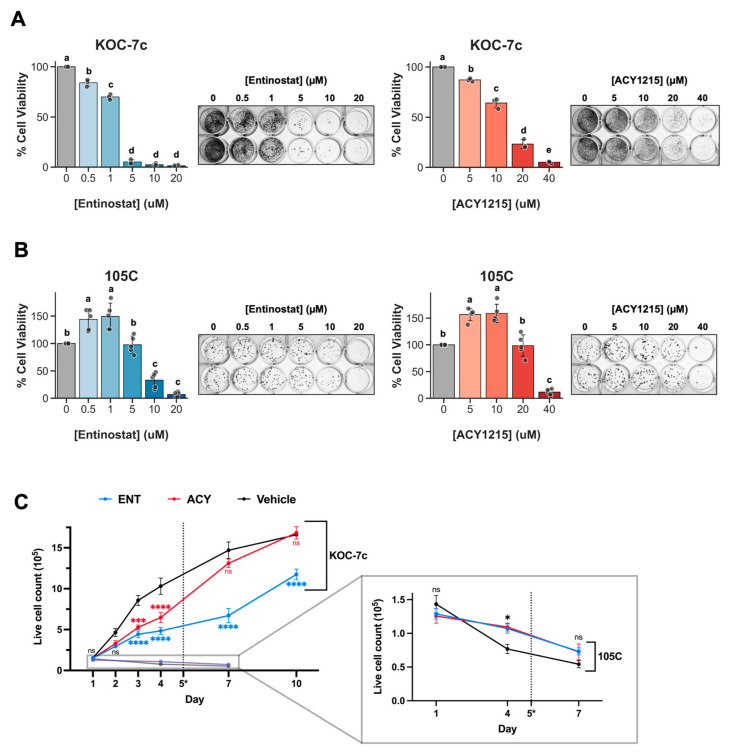
Dormant and proliferative OCCC spheroids have opposing responses to HDACi treatment. (**A**,**B**) Reattached OCCC cell line spheroids show differential sensitivity to HDACi. KOC-7c and 105C cells were seeded at 25 K and 100 K cells/well, respectively, in 24-well ULA culture and treated with various concentrations of ENT and ACY at the time of seeding. After 3 days, all spheroid cells and media were transferred, well-by-well, into 24-well adherent culture plates. Each well was also supplemented with 500 µL fresh media at this time. Cells were allowed to adhere to adherent culture plates for 24–48 h. AlamarBlue™ cell viability reagent was used to quantify treatment response. Then, all wells were stained with HEMA 3 to visualize colonies from attached spheroids (adjacent panels). One-way ANOVA followed by Tukey’s multiple-comparison test was conducted. Different letters (a, b, c, d, and e) represent significantly different values (*p* < 0.05). (**C**) KOC-7c and 105C spheroid viability over time after HDACi treatment. Cells were seeded at 100 K cells/well in 24-well ULA and treated with 1 µM ENT, 5 µM ACY, or vehicle at the time of seeding. Cells were given a single dose and then supplemented with fresh media on day 5 (denoted by doted vertical lines and *). Trypan blue exclusion cell counting was performed at every time point. Two-way ANOVA was conducted, followed by Dunnett’s multiple-comparison test comparing treated groups to vehicle for each time point. *p*-value summary: >0.05 (ns), ≤0.05 (*), ≤0.001 (***), and ≤0.0001 (****); black labels correspond to both treatment groups, while blue and red labels correspond to ENT and ACY, respectively.

Next, we employed a spheroid reattachment assay to further assess and compare the viability of KOC-7c and 105C spheroids under ENT and ACY treatment. The assay requires viable spheroid cells to retain the ability to reattach onto cell culture substratum, providing a robust and stringent assessment of cell viability, and we hypothesized that HDACi-induced changes to the epigenome may compromise the ability of spheroid cells to reattach to an adherent surface. This also works to mimic the formation of secondary peritoneal lesions during OCCC metastasis in vitro. There was a dose-dependent decrease in KOC-7c spheroid cell viability upon treatment with both inhibitors (Figure 8A). An ENT concentration of 5 µM and ACY concentration of 40 µM resulted in a maximal reduction in KOC-7c spheroid cell viability (Figure 8A). Surprisingly, 105C spheroids treated with 1–5 µM ENT and 5–10 µM ACY had a significantly higher cell viability compared to the vehicle control, and 105C spheroid viability did not significantly decrease until 10 µM ENT and 40 µM ACY (Figure 8B). This increase in 105C spheroid viability was also replicated with low doses of Panobinostat (1.5–24 nM) and Belinostat (150–1800 nM) (Figure S2), but not when 105C spheroids were treated after 3 days in suspension culture (Figure S3), showing that this unique resistance is likely not due to inhibition of specific HDAC proteins and occurs only when HDACs are inhibited during early spheroid formation.

To determine the response of KOC-7c and 105C spheroids to HDAC inhibition over time, we performed trypan blue exclusion cell counting on spheroids over a treatment period of 1.5 weeks (Figure 8C). Trypan blue cell counting quantifies raw spheroid cell number and provides values comparable over many time points, as opposed to alamarBlue™, which uses relative fluorescence as its measure of cell viability. KOC-7c and 105C cells were treated with a single dose of 1 µM ENT or 5 µM ACY at the time of seeding to ULA culture, and cells were counted at each indicated time point. KOC-7c cells continued to proliferate under treatment with both inhibitors, albeit at a slower pace compared to the vehicle. Both ENT and ACY treatment significantly reduced live cell counts of KOC-7c spheroid cells on days 3 and 4. ENT-treated KOC-7c spheroids consistently had significantly lower cell counts than vehicle-treated cells after day 2, while ACY-treated spheroids reached cell counts similar to the vehicle treatment by day 7, following the addition of fresh media on day 5, likely because cells regain their proliferative abilities due to drug dilution and breakdown in culture (Figure 8C). Overall, a single dose of either ENT or ACY can slow KOC-7c spheroid cell growth for up to a week.

For 105C spheroids, ENT and ACY did not result in differences in cell count after 1 day of treatment. While spheroids of all three treatment groups experienced a reduction in total cell number over time, both ENT- and ACY-treated spheroids had a higher cell count compared to the vehicle control on day 4, which was no longer evident by day 7 (Figure 8C). Day 3 ENT-treated 105C spheroid cell counts, pooled from several separate experiments, also show a statistically significant increase in cell count (Figure S4). Western blot analyses of ENT-treated 105C spheroids exhibited lower levels of cleaved caspase 3, an apoptosis marker, suggesting intrinsic resistance to ENT by protection from detachment-related cell death (Figure S5). Collectively, these results suggest that the increase in cell viability seen in 105C spheroids treated with HDACi (Figure 8B) was due to an increase in total cell number through reduced cell death and not a product of the reattachment process. This is also a transient phenotype that is reversed by day 7.

### 3.5. Effects of Entinostat Treatment on Spatial Localization of Histone Modifications and Proliferation Marks in OCCC Spheroids

**Figure 9:**
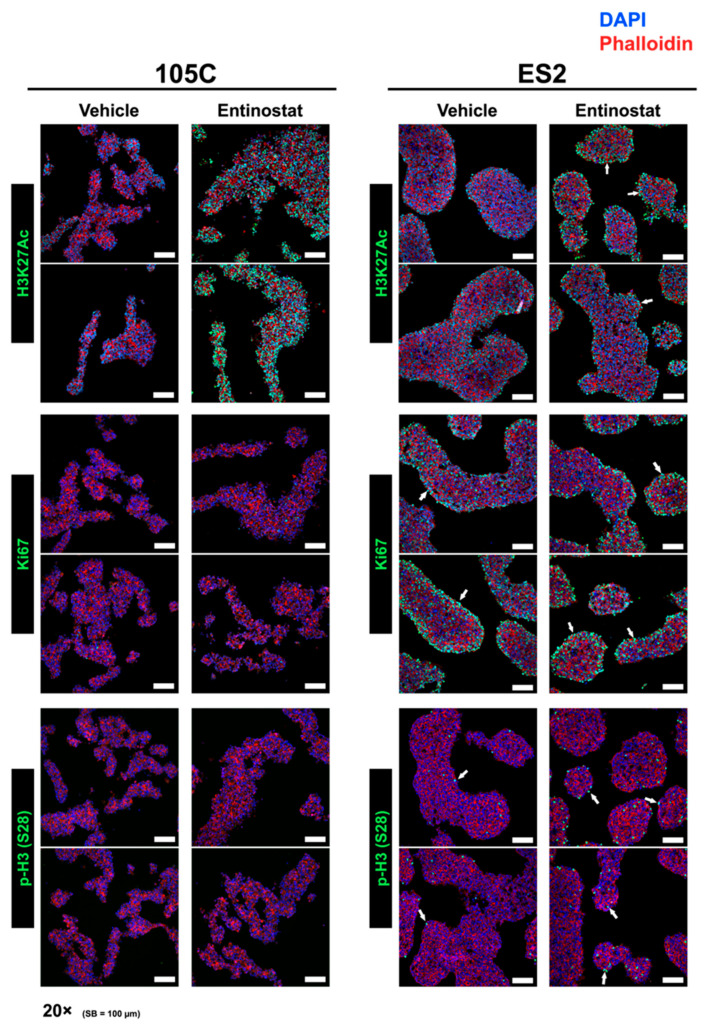
Effects of Entinostat treatment on localization of histone post-translational modifications within spheroid structures. 105C and ES2 cells were seeded in 6-well ULA and treated with 1 µM Entinostat for 3 days. Spheroids were collected and embedded in Cryomatrix optimal cutting temperature compound, sectioned to 5 µm, and then mounted on microscope slides for immunofluorescence staining for H3K27Ac, Ki67, and phosphorylated Histone 3 (S28). Stained sections were visualized using the Leica DMI4000 B Automated Inverted Microscope (Richmond Hill, ON, Canada) equipped with a Leica K5 sCMOS camera. Representative images were taken at 20× magnification. White arrows indicate H3K27Ac, Ki67 or phosphorylated Histone 3 (S28) in the nuclei of peripheral cells in ES2 spheroids. Scale bar = 100 µm.

To investigate differences in the spatial localization of histone modifications in proliferating and dormant spheroids upon HDACi treatment, we performed immunofluorescence imaging for histone modifications on 105C and ES2 spheroid cryosections. The ES2 cell line was used to model proliferating spheroids instead of KOC-7c spheroids given that the ES2 line produces compact spheroids in contrast to the KOC-7c cell line, for which its spheroids lack a distinct 3D structure, which presents difficulties during cryosectioning, staining, and data interpretation. As expected, Entinostat treatment elevated H3K27Ac staining in both 105C and ES2 spheroids. H3K27Ac staining was uniform across 105C spheroid structures but was localized to the periphery in ES2 spheroids (Figure 9). We also assessed whether spheroid cell proliferation was altered upon HDACi treatment and whether actively proliferating cells co-localize with histone post-translational modifications in spheroids. Spheroid cryosections were stained for antigen Kiel 67 (Ki67) and phospho-histone H3 at Ser28 (p-H3) as markers of proliferation and mitotic entry, respectively (Figure 9). 105C spheroids showed minimal staining for either marker regardless of treatment. Ki67 and p-H3 staining were localized at the periphery of ES2 spheroids, and there was no change in staining intensity after treatment by qualitative assessment. Together, this shows that Entinostat treatment targets almost all cells in a 105C spheroid but mostly affects peripheral, actively cycling cells in ES2 spheroids.

### 3.6. Treatment of OCCC Cells Prior to Spheroid Formation Enhances HDACi Sensitivity in Spheroids on a Cell-Line-Dependent Basis

**Figure 10:**
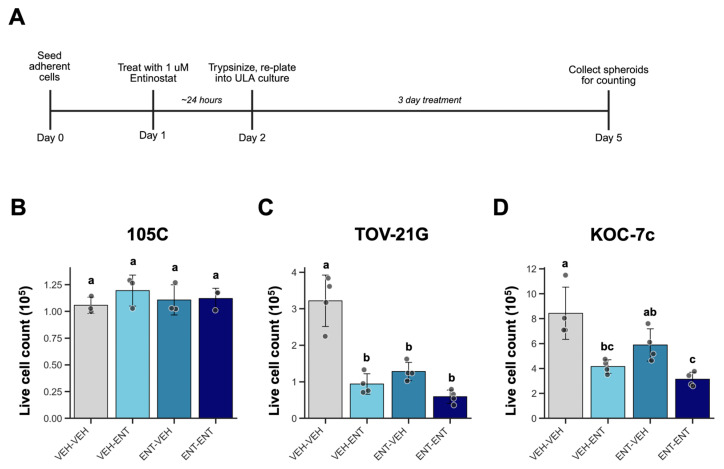
Effects of Entinostat pre-treatment on OCCC spheroid viability. (**A**) Schematic showing treatment regime and time-course of experiments. 105C (**B**), TOV-21G (**C**), and KOC-7c (**D**) monolayer cells were treated with 1 µM Entinostat or vehicle for 24 h; then, cells were replated into 24-well ULA at 25–100 K cells/well and treated with 1 µM Entinostat or vehicle for 3 days. Trypan blue exclusion cell counting was performed after the treatment period. One-way ANOVA followed by Tukey’s multiple-comparison test was conducted. Different letters (a, b and c) represent significantly different values (*p* < 0.05) (n = 3 105C; n = 4 TOV-21G, KOC-7c).

Given that H3K27Ac levels are regulated both temporally and spatially in OCCC spheroids, we sought to determine if elevating H3K27Ac in OCCC cells with HDACi prior to seeding to suspension culture may interfere with spheroid formation and viability. To this end, we treated OCCC cell lines 105C, KOC-7c, and TOV-21G monolayer cells with Entinostat for 24 h prior to treatment with ENT as spheroids and assessed the live cell count after 3 days (Figure 10A). Like KOC-7c, TOV-21G cells also harbor classic OCCC mutations and proliferate as spheroids. Treatment groups are named as “pre-treatment –treatment in spheroid” (i.e., vehicle (VEH) pre-treatment + VEH treatment in spheroid is named “VEH—VEH”). For 105C cells, there was no difference in live spheroid cell count between all four treatment groups (Figure 10B). There was a small increase in cell number in ENT-treated cells of both pre-treatment groups, but this was not statistically significant. In TOV-21G cells, ENT—VEH spheroids had a significantly lower cell count compared to VEH—VEH spheroids, meaning that a single ENT dose prior to spheroid formation was sufficient to reduce survival/proliferation as a spheroid (Figure 10C), and as expected, both ENT—VEH and ENT—ENT spheroids also had a significantly lower cell count compared to VEH—VEH cell counts. Proliferative KOC-7c spheroids showed a very similar trend in cell counts to TOV-21G spheroids (Figure 10D). Together, this shows that elevated histone acetylation prior to spheroid formation impedes spheroid viability to different extents in OCCC cell lines and alludes to different dependencies on histone acetylation for early stages of anchorage-independent growth.

### 3.7. RNA-Seq Analysis Reveals Distinct Transcriptomic Responses to Entinostat in Dormant and Proliferative OCCC Spheroids

**Figure 11:**
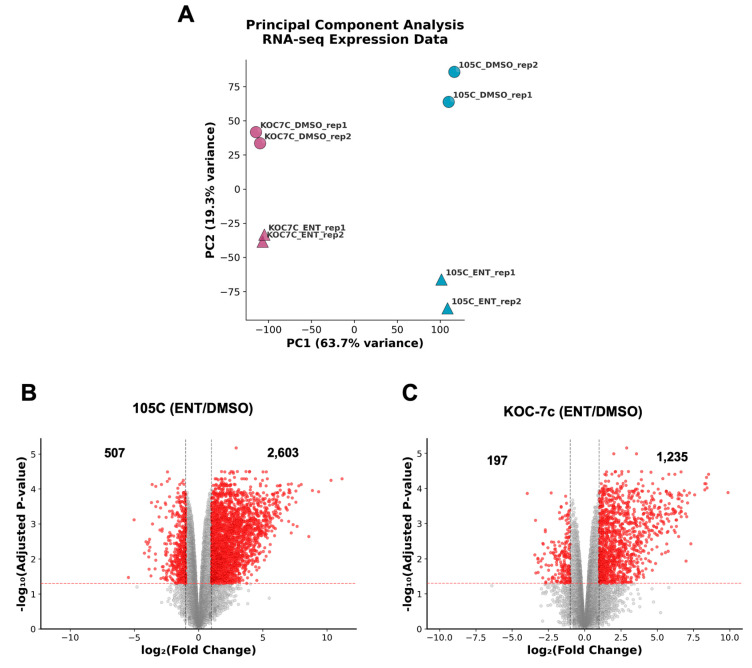
RNA-Seq analysis of 105C and KOC-7c cell line spheroids, treated with Entinostat or DMSO. (**A**) PCA plot on RNA-Seq data showing the distinct grouping of 105C and KOC-7c cell lines and further separation between treatments within each group, indicating that the largest differences between the samples are primarily due to cell types, with further differences attributed to Entinostat treatment. (**B**,**C**) Volcano plots of RNA-Seq data for both 105C and KOC-7c cell lines (ENT/DMSO) (*p* < 0.05 and absolute (fold change) > 2). The number of genes with expressions significantly altered by ENT treatment is indicated in each quadrant for each cell line. Grey and red-dashed lines indicate thresholds used for fold-change and adjusted *p*-value, respectively.

To elucidate the molecular mechanisms underlying the differential responses of dormant and proliferative OCCC spheroids to HDAC inhibition, we performed RNA-seq on 105C and KOC-7c spheroids treated with Entinostat (1 µM) or vehicle control (DMSO) for 3 days. Principal component analysis (PCA) revealed that cell line identity was the primary driver of transcriptomic variance, with 105C and KOC-7c samples clustering distinctly along the first principal component (Figure 11A). Within each cell line cluster, Entinostat-treated and vehicle-treated samples segregated along the second principal component, demonstrating that HDAC inhibition induced reproducible and cell line-specific transcriptional changes. Notably, there was greater segregation between Entinostat- and vehicle-treated 105C spheroids than KOC-7c spheroids across the second principal component, suggesting that Entinostat alters the 105C spheroid transcriptome more than that of KOC-7c spheroids.

Differential gene expression analysis revealed markedly different transcriptional responses to Entinostat between the two cell lines. In dormant 105C spheroids, Entinostat treatment resulted in substantial transcriptomic alterations, with a pronounced asymmetry favoring gene upregulation, based on the relative number of genes upregulated by ENT in 105C spheroids relative to KOC-7c spheroids (Figure 11B). This is consistent with the canonical mechanism of HDAC inhibition, whereby the maintenance of histone acetylation promotes a more permissive chromatin state and transcriptional activation. Proliferative KOC-7c spheroids also exhibited a similar dynamic (asymmetrical upregulation of genes); however, far fewer genes were significantly impacted relative to 105C (KOC-7c: 1432 genes up- or downregulated vs. 105C: 3110) (Figure 11C). These findings suggest that the transcriptional consequences of HDAC inhibition are blunted in the KOC-7c vs. 105C.

### 3.8. Gene Set Enrichment Analysis Reveals Broad Transcriptional Reprogramming upon HDAC Inhibition in 105C and KOC-7c Spheroids

**Figure 12:**
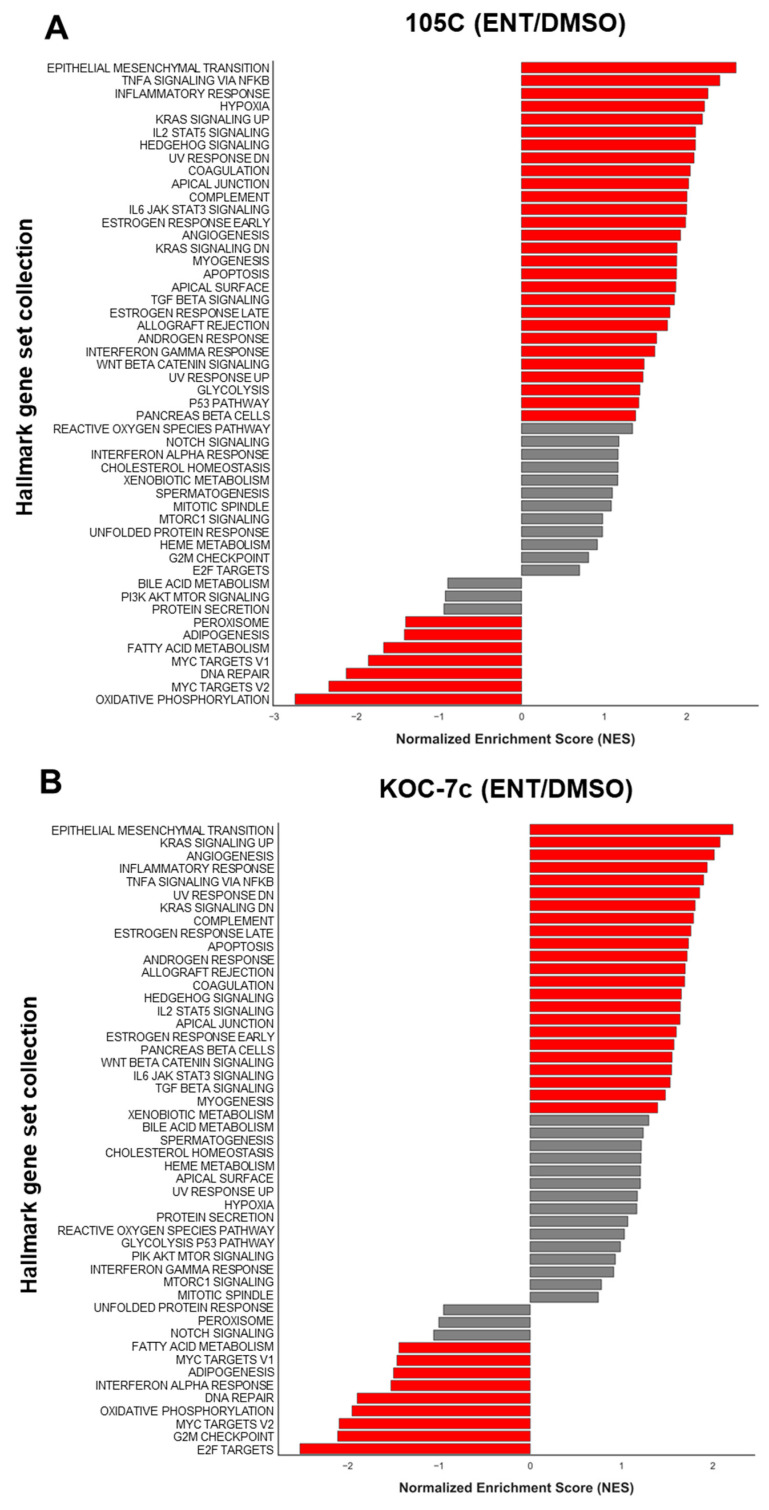
GSEA analyses of differentially expressed genes in (**A**) 105C and (**B**) KOC-7c lines reveal pathways downregulated when cells are grown in suspension as 3D spheroids in the presence of Entinostat (red bars: FDR-adjusted *p*-value < 0.05).

To identify biological pathways and processes modulated by Entinostat treatment, we performed gene set enrichment analysis (GSEA) on the differentially expressed genes within each cell line. HDAC inhibition induced widespread transcriptional changes affecting multiple cancer hallmark pathways in both 105C and KOC-7c spheroids, with pathways showing both significant upregulation and downregulation (Figure 12A,B). Surprisingly, many of the same pathways were significantly up- and downregulated between the two cell lines. Epithelial-to-mesenchymal transition, inflammatory signaling, and KRAS and TGFb signaling pathways were among the shared upregulated pathways, while MYC signaling and metabolic pathways were downregulated. Only 5/35 (105C) and 4/32 (KOC-7c) significantly altered pathways were unique to that cell line (Figure 12). Notably, the G2/M checkpoint and E2F targets were significantly downregulated in KOC-7c spheroids after Entinostat treatment but were not significantly altered in 105C spheroids. This supports the Entinostat-induced growth arrest in KOC-7c spheroids and elevated viability in 105C spheroids.

While this within-cell-line analysis demonstrates that Entinostat elicits substantial transcriptional reprogramming in both cell line spheroids, it is important to recognize the inherent limitations of comparing separate GSEA analyses between cell lines. A pathway identified as significantly downregulated in one cell line may also be downregulated in the other cell line, but to a markedly different degree; such quantitative differences would not be apparent from independent GSEA analyses. Consequently, while both 105C and KOC-7c spheroids show alterations in overlapping pathway categories, the magnitude and biological significance of these changes cannot be meaningfully compared using this approach. To address this limitation and directly interrogate the differences in Entinostat response between dormant and proliferative spheroids, we next performed a comparative analysis using the ratio of gene expression changes between the two cell lines.

### 3.9. Direct Comparison of Entinostat Responses Reveals Cell-Line-Specific Transcriptional Programs

To overcome the limitations of comparing independent GSEA analyses and directly quantify differences in Entinostat response between dormant and proliferative spheroids, we employed a comparative analytical approach. For each gene, we calculated the ratio of expression changes: [105C(ENT/DMSO)]/[KOC-7c(ENT/DMSO)]. This ratio identifies genes where the magnitude or direction of Entinostat-induced changes differs between the two cell lines, enabling meaningful between-line comparisons that are not possible with separate within-line analyses.

**Figure 13:**
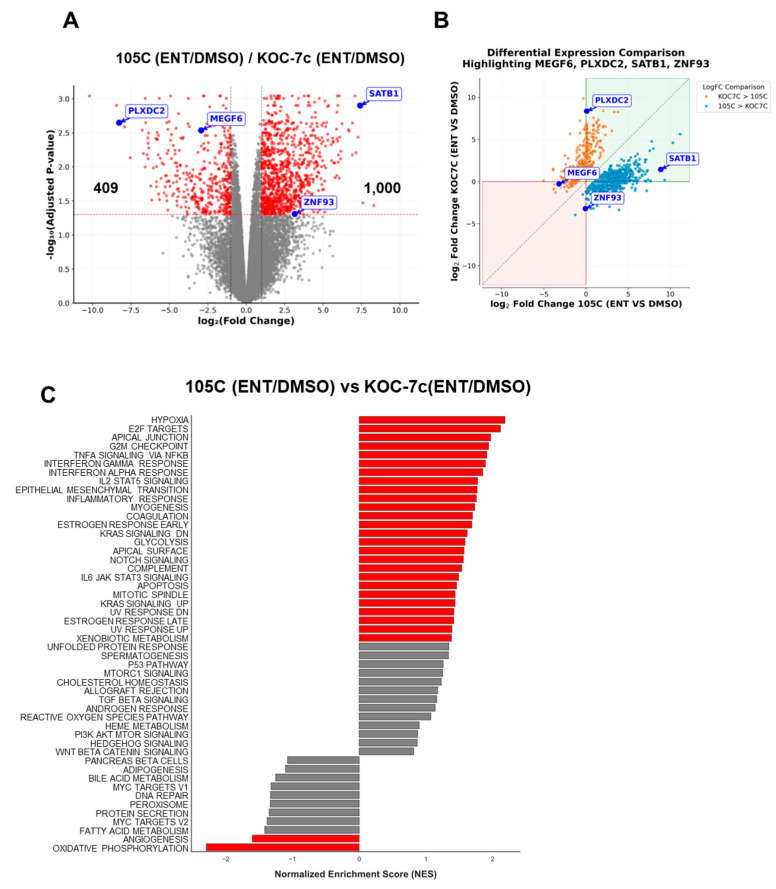
Identifying differential responses to Entinostat between 105C and KOC-7c spheroids based on RNA-seq data. (**A**) Volcano plots of RNA-Seq data 105C (ENT/DMSO) vs. KOC-7c (ENT/DMSO). *p* < 0.05 and absolute (fold change) > 2). Grey and red-dashed lines indicate thresholds used for fold-change and adjusted *p*-value, respectively. (**B**) Comparison of Entinostat-versus-control (DMSO) gene expression changes between the 105C and KOC-7c cell lines. Each point represents a gene, plotted as the fold change (ENT/DMSO) in 105C vs. the fold change (ENT/DMSO) in KOC-7c; genes shown are significantly different between KOC-7c (ENT/DMSO) and 105C (ENT/DMSO). Genes significantly upregulated in KOC-7c relative to 105C (based on ENT/DMSO ratios) are shown in orange, and genes significantly downregulated are shown in blue. The red and green quadrants denote genes with concordant direction of regulation in both cell lines (i.e., decrease in both or increase in both, respectively), where differences in the magnitude of ENT/DMSO change are sufficient to yield significant differential regulation between KOC-7c and 105C. The white quadrants indicate genes regulated in opposite directions between cell lines, consistent with predominantly cell line-specific responses. The spotlighted genes (blue in the white box) correspond to genes in panel (**A**) that show different expression dynamics. Grey dashed line shows where x = y. (**C**) GSEA signatures identified as being differentially expressed between lines in response to Entinostat treatment.

Differential expression analysis using this comparative approach revealed a substantial number of genes with significantly different responses to Entinostat between 105C and KOC-7c spheroids (Figure 13A). To visualize the direction and magnitude of these differential responses, we plotted the fold change (Entinostat/DMSO) in 105C against the corresponding fold change in KOC-7c for each differentially expressed gene (Figure 13B). Genes clustering in the concordant quadrants (green and red) represents those regulated in the same direction in both cell lines but with significantly different magnitudes—precisely the type of difference that would be missed by comparing separate GSEA analyses. Genes in the discordant quadrants (white) indicate cell-line-specific responses with opposing directions of regulation. Strikingly, a substantial proportion of differentially expressed genes exhibited cell-line-specific responses, with many genes being upregulated upon Entinostat treatment in 105C spheroids but downregulated or unchanged in KOC-7c spheroids and vice versa. We highlighted four genes (SATB1, ZNF93, PLXDC2, and MEGF6) to illustrate where they fell when compared as ratios between the 105C and KOC-7c cell lines. This pattern confirms that HDAC inhibition activates distinct transcriptional programs in dormant versus proliferative spheroids.

We then performed GSEA on the ranked list of genes based on their differential response between cell lines, which identified pathways preferentially modulated in one cell line relative to the other upon Entinostat treatment (Figure 13C). This approach directly addresses the question of which biological processes respond differently to HDAC inhibition depending on the spheroid phenotype. Most notably, the G2/M checkpoint pathway emerged as being significantly enriched among genes showing greater upregulation in 105C spheroids compared to KOC-7c spheroids in response to Entinostat. This finding is particularly relevant given the divergent proliferative phenotypes of these cell lines and suggests that HDAC inhibition preferentially de-represses cell cycle regulatory genes in dormant spheroid cells.

### 3.10. Entinostat Treatment Derepresses G2/M Checkpoint Genes Specifically in Dormant 105C Spheroids

**Figure 14:**
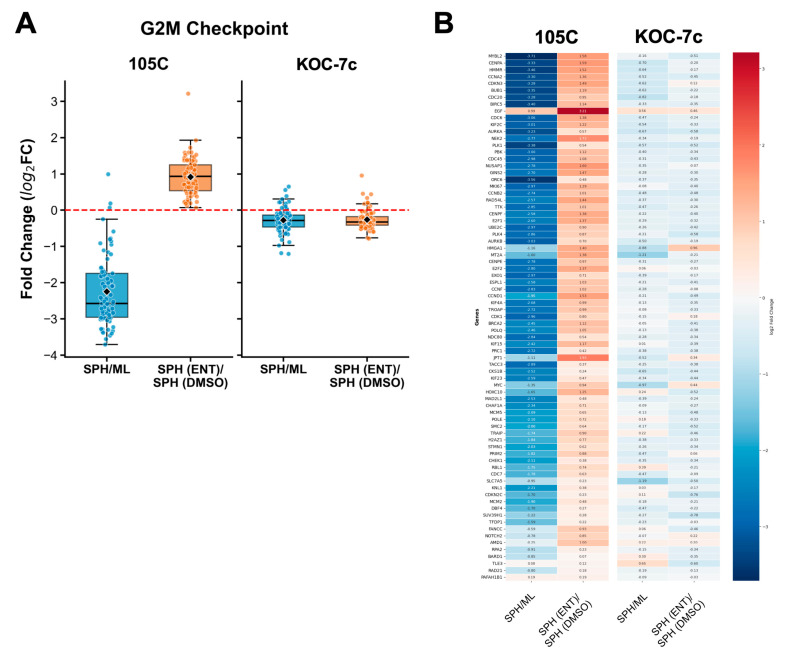
Enrichment of the GSEA-defined G2/M checkpoint signature in 105C spheroids in response to Entinostat is driven by derepression of core enrichment genes. (**A**) The core enrichment genes responsible for the differential G2/M checkpoint signature between 105C (ENT/DMSO) and KOC-7c (ENT/DMSO) are downregulated in 105C (SPH/ML) and are upregulated in response to Entinostat treatment in 105C (SPH(ENT)/SPH(DMSO)). These genes remain unchanged in either KOC-7c (SPH/ML) or KOC-7c (SPH(ENT)/SPH(DMSO). Black diamond indicates the mean log_2_FC of all genes in group. (**B**) Heat map and fold change of individual core enrichment genes within the GSEA G2/M checkpoint gene set.

Given the significant enrichment of the G2/M checkpoint signature among genes differentially regulated between 105C and KOC-7c spheroids upon Entinostat treatment, we sought to characterise the expression dynamics of the core enrichment genes driving this signature. Analysis of these genes revealed that they were substantially downregulated in 105C cells during the transition from monolayer to spheroid culture (Figure 14A, SPH/ML comparison) [38]. This downregulation is consistent with the dormant, non-proliferating phenotype of 105C spheroids and reflects the suppression of cell cycle progression genes during the establishment of quiescence.

Remarkably, Entinostat treatment resulted in the upregulation of the same G2/M checkpoint genes in 105C spheroids (Figure 14A, SPH(ENT)/SPH(DMSO) comparison), effectively reversing the transcriptional repression that occurred during spheroid formation. This de-repression was not observed to the same extent in KOC-7c spheroids, which maintain expression of cell cycle genes during anchorage-independent growth. The heat map visualisation of individual core enrichment genes confirmed that key regulators of the G2/M transition, including cyclins and checkpoint kinases, were significantly upregulated in 105C spheroids upon Entinostat treatment (Figure 14B). Without ChIP-seq data to clearly demonstrate that HDACi treatment increases the level of H3K27Ac in the regulatory regions of G2/M checkpoint genes, we cannot conclude that this is the mechanism by which 105C spheroid cells maintain a proliferative phenotype within the first 3 days of spheroid formation. G2/M checkpoint gene products are primarily controlled by post-translational modifications such as phosphorylation; therefore, there are a myriad of mechanisms that could explain the phenotype we observed, including indirect upstream consequences related to the activity of cell cycle regulatory proteins, which change the activity of G2/M proteins through changes in phosphorylation state [72,73,74,75,76].

## 4. Discussion

The present study sought to characterize the epigenetic dynamics within OCCC spheroids, specifically the reliance on histone acetylation, using HDACi Entinostat and ACY-1215 as the agents for investigation. The comparison of the KOC-7c and 105C OCCC cell lines was integral to our investigation, as they share the classic OCCC gene mutation profile but have vastly different abilities to proliferate as spheroids. OCCC commonly presents with ARID1A-loss, resulting in profound genome-wide epigenetic changes, especially regarding H3K27Ac levels and HDAC function. There is limited understanding of EOC spheroid biology and the mechanisms for sustaining viability in an anchorage-independent state, especially from an epigenetic perspective. As previous studies have shown, EOC spheroids are composed of a collection of both proliferative and dormant cells [54,77,78,79,80]; an understanding of the epigenetic vulnerabilities of both populations fills a significant gap in knowledge that ultimately leads to the identification of targeted therapies. To our knowledge, this is the first time that the effects of HDAC inhibition have been extensively compared between proliferative and dormant OCCC spheroids using relevant in vitro models.

The observation that global H3K27Ac levels change during early spheroid formation in several OCCC cell lines (Figure 2) implies that acetylation dynamics are regulated during spheroid formation and are potentially important for their viability in a non-adherent state. This corroborates our interest in investigating histone acetylation dynamics during EOC metastasis. In five OCCC cell lines, two were non-proliferative as spheroids (105C and EFO-27) and showed varying trends in H3K27Ac levels in the spheroid over time, suggesting that the role of H3K27Ac on spheroid biology is cell-line-specific. Cancer cells can use lactate, a byproduct of high glycolytic activity, as the carbon source for histone acetylation [77]. In addition, histone acetylation has been shown to respond rapidly to metabolic changes, and global acetylation levels decrease in response to quiescence [78]. Interestingly, the only two cell lines with a reduction in H3K27Ac over time, 105C and EFO-27, were also the only cell lines that were dormant as spheroids and did not deplete their media. A hallmark characteristic of cell dormancy is low metabolic activity [79], which reduces the availability of metabolic intermediates, namely, acetyl-CoA, needed for HAT activity. Thus, the reduction in H3K27Ac in dormant OCCC spheroids may reflect reduced metabolic activity, which negatively impacts HAT activity, and the continued HDAC activity results in decreased H3K27Ac, which was demonstrated in a previous study in transcriptionally inactive cells [80]. Together, these time course experiments show that H3K27Ac is uniquely regulated in each OCCC cell line during their transition from adherent to spheroid conditions.

The distinct spatial localization patterns of H3K27Ac and proliferative marks in OCCC spheroids provide further insight into the epigenetics of spheroid biology. The ES2 cell line forms dense spheroids that represent the prototypical 3D spheroid structure with distinct nutrient and oxygen concentration gradients from the surface to core; the resulting structure has a proliferative outer layer, with both H3K27Ac and cell proliferation marks being localized here, and a dormant core that could be hypoxic and necrotic [81,82,83]. All cells within an ES2 spheroid likely have a propensity for anchorage-independent growth, and it is the delegation of cells to either the periphery or the core that determines an individual cell’s proliferative capacity. Thus, the ES2 cell line provides a unique model for investigating H3K27Ac dynamics in EOC spheroids since there are both dormant and proliferating cells within the same structure. Since cells are treated at the time of seeding to suspension, cells are exposed to the drug as they begin to aggregate into spheroids. Therefore, the low staining for H3K27Ac in the core of ES2 spheroids after ENT treatment was surprising and suggests that the spatial orientation of cells within the spheroid core is the main contributor to H3K27Ac downregulation, which could be indicative of dormancy. This also supports H3K27Ac downregulation in dormant cells as a likely consequence of inefficient HAT activity, as HDAC inhibition could not recover H3K27Ac levels in the core. On the other hand, proliferation marks and H3K27Ac were undetectable in dormant 105C spheroids in the absence of ENT treatment, and 105C spheroids lacked spatial heterogeneity, highlighted by strong H3K27Ac staining throughout the entire spheroid upon ENT treatment. This suggests that, as opposed to ES2 spheroids, the onset of 105C spheroid dormancy is not induced by spheroid structure, and this cell line has intrinsic biology incompatible with anchorage-independent growth. This is a qualitative assessment based on IF intensity and localization. Further experiments can employ quantitative IF detection from multiple OCCC cell line spheroids to provide more definitive data on the spatial localization of H3K27Ac marks within spheroid structures.

Using HDAC inhibitors, we showed that H3K27Ac is differentially regulated in KOC-7c and 105C spheroids. Proliferating KOC-7c monolayer and spheroid cells were sensitive to HDACi treatment. Spheroids exhibited slowed growth and elevated apoptosis, suggesting that regulation of H3K27Ac dynamics is required for viability and efficient proliferation in both culture conditions. Similarly, all 16 OCCC cell lines cultured in monolayer (i.e., proliferative) were sensitive to HDACi treatment, and although the degree of sensitivity varied, this shows that reliance on H3K27Ac for proliferation is widely applicable across actively cycling cells, which is supported by ChIP-seq studies showing that H3K27Ac plays a crucial role during cell division by “bookmarking” cell-identity-related genes for rapid transcriptional reactivation during M-to-G1 transition [84,85]. In contrast, dormant 105C spheroids were not only impervious to all HDAC inhibitors tested but also displayed elevated viability at lower relative concentrations with decreased apoptotic marker expression. Moreover, others have shown that HDACs can regulate checkpoint kinase phosphorylation and can downregulate the activity of the AKT signalling pathway, which in turn decreases expression of checkpoint kinases such as p-WEE1 [86,87]. This implies that downregulating H3K27Ac levels is important for entry into a dormant state during early spheroid formation. In support, studies in both yeast and mammalian cells have found that histone acetylation levels are decreased in dormant cells, and regulation of acetylation levels is critical for the entry and exit from dormancy [88,89,90]. However, the gradual decrease in spheroid cell number over the treatment period (Figure 8C) and the fact that ENT treatment after the onset of spheroid dormancy did not elicit the same increase in viability (Figure S3) suggest that elevating H3K27Ac is insufficient for conferring long-term anoikis resistance in 105Cs and is unsuccessful at reversing their dormant state. Together, these results show that HDACi treatment elicits different responses in dormant spheroids depending on the quiescence status at the time of treatment, but overall, this phenotype confers resistance to HDACi, while proliferating spheroids are sensitive, likely due to their reliance on H3K27Ac for active proliferation.

ENT pre-treatment experiments tested the necessity of proper H3K27Ac regulation during the transition from adherent to spheroid culture. ENT pre-treated 105C spheroids showed no changes to cell number, while a single 24 h dose of ENT prior to spheroid formation reduced TOV-21G and KOC-7c spheroid cell count by more than half. This data shows that dormant and proliferative spheroids have different dependencies on H3K27Ac regulation during early spheroid formation. As cells transition from a proliferative monolayer to a spheroid culture, they undergo dramatic cellular reprogramming to retain viability, and they clearly have different dependencies on histone acetylation to do so. Cellular stress, metabolic rewiring, dormancy, and histone acetylation are intrinsically linked [88]. The lack of cell adhesion may induce intracellular stress pathways in 105C cells, which induce dormancy and decrease H3K27Ac and transcriptional activation; increasing H3K27Ac via ENT treatment helps maintain viability of spheroids in the short term, as it may transiently counteract the onset of dormancy by maintaining genomic acetylation levels. Dormant cells also have lower levels of transcription [91], and accordingly, previous data showed that several cell cycle regulators are downregulated in 105C spheroids [38]. Sources also suggest that disruption of epigenetic regulators prevents cancer cells from activating the necessary transcriptional programs to elicit adaptive stress responses in unfavorable environments, thus delaying apoptotic programming [92].

Our RNA-seq data showed that Entinostat elicits comparable transcriptional programs in 105C and KOC-7c spheroid cells, which suggests many shared HDAC targets between cell lines. Despite this, the cell line spheroids show opposing phenotypic responses to treatment, suggesting that their distinct biological backgrounds dictate sensitivity to HDACi. From our previous RNA-seq data, 105C spheroids downregulate G2/M checkpoint proteins when compared to monolayer cells [38]; here, we show the derepression of G2/M checkpoint genes after Entinostat treatment in 105C spheroids (Figure 14), which also corroborates the distinct but transient protection from cell death in 105C spheroids (Figure 8B,C). This suggests that the dormancy program in 105C spheroids involves epigenetic regulation of the G2/M checkpoint pathway. However, given that the elevation in 105C spheroid viability is transient, this indicates that transcriptional de-repression alone is insufficient for driving these dormant cells back into active proliferation but can maintain a transcriptionally active state that may delay full commitment to dormancy. In KOC-7c spheroids, elevated genomic histone acetylation impacts the G2/M checkpoint and E2F targets, which results in growth inhibition and cell death, in stark contrast to 105C spheroids. Together, these data reveal that the differential sensitivity of dormant and proliferative OCCC spheroids to HDAC inhibition is underpinned by distinct transcriptional responses, with dormant spheroids exhibiting derepression of cell cycle genes that do not translate into renewed proliferation over the long term. The negative effects of the imbalance in H3K27Ac throughout the genome overwhelm the proliferative response in 105C spheroid cells, eventually leading to a loss in cell viability. Consequently, the widespread elevated levels of H3K27Ac deposition due to HDAC inhibition cannot be overcome regardless of the spheroid cell proliferative program.

While our work strived to maximize therapeutic relevance and translational potential using relevant 3D culture vessels to model spheroids and interrogating a wide range of OCCC cell lines to capture possible interpatient heterogeneity, in vitro work often fails to capture the complexity of tumor biology. Drug response is heavily influenced by ECM structure, autocrine and paracrine signaling, vasculature, and cell–cell interactions, which standard tissue culture fails to accurately model. The IC50s of HDACis determined in this work cannot reflect the doses required to achieve a tumor-regressive phenotype in a patient. PDX experiments would be necessary to determine doses that reduce tumor size and growth and increase apoptosis in a murine model. However, these models are immunodeficient and therefore likely do not provide an accurate dosing regimen for a patient population. The literature shows that MS-275 (Entinostat), in a Phase 1 clinical trial, requires a dose of 4–6 mg/m^2^ biweekly to achieve a plasma concentration of 4.6 nM over a 7-day period [93]. Similar studies for ACY-1215 using a plasmacytoma mouse model showed that the plasma level of ACY was 246.8 nM after 24 h [94]. Our in vitro studies revealed that ENT and ACY had IC50 concentrations of 0.83 µM to 15.5 µM, which were higher than those observed in the above publications, emphasizing the need for in vivo studies to determine optimal pharmacokinetic data. Future studies will implement PDX and organoid models, which take into consideration the ways that resident CAFs and immune cell infiltration impact HDACi efficacy [95,96,97,98,99,100,101]. It is also critical to note that ambient oxygen levels (21% O_2_) are much higher than tissue oxygen levels (~5% O_2_) [102,103]. Given that histone acetylation is heavily influenced by metabolic activity [88,91,104,105,106], which is largely dictated by oxygen availability, future work may look to control experimental oxygen conditions to improve data interpretability. Furthermore, knowledge on the architecture of patient spheroids and ascites fluid composition would be critical for improving current in vitro model systems, but this is currently an under-researched area.

Overall, our study investigated the utility and functional consequences of HDACi treatment on OCCC spheroid biology and the H3K27Ac dynamics during OCCC metastasis. We sought to gain a deeper understanding of how an HDAC inhibitor such as Entinostat alters the biology of OCCC cell line spheroid cells, especially between proliferative and dormant spheroids, using the 105C and KOC-7c cell lines, which display these spheroid phenotypes. We revealed that H3K27Ac levels are differentially regulated in KOC-7c and 105C spheroids, which allow spheroids to take on a proliferative or dormant phenotype, respectively; the maintenance of H3K27Ac levels in KOC-7c is likely important for proliferation, while downregulation is important for 105C dormancy. These inherent epigenetic differences determine the sensitivity of KOC-7c and the resistance of 105C spheroids to H3K27Ac-altering HDACi treatment. Mechanistically, HDACi prevents downregulation of H3K27Ac during 105C spheroid formation, which may prevent the onset of dormancy and stress response activation through “phenotypic inertia” [92], resulting in an elevated viability. Therapeutics that prevent or reverse the onset of dormancy could be considered for combination treatment with HDACi to target the dormant spheroid phenotype [107]. From a translational standpoint, our results suggest that the utility of HDACi in OCCC is highly context-dependent. HDACi is potentially effective as a targeted therapy against proliferative spheroid survival, but other therapeutic approaches should be considered for dormant spheroids, as their phenotype confers resistance.

## Figures and Tables

**Table 1:** Antibodies used in immunoblotting experiments.

Antibody	Company	Catalogue #
Alexa Fluor 488-conjugated anti-rabbit IgG	ThermoFisher Scientific	A48282
Actin	MilliporeSigma	A2066
Anti-mouse IgG	MilliporeSigma	NA931V
Anti-rabbit IgG	MilliporeSigma	NA934V
Caspase 3 (cleaved)	Cell Signaling Technology (Danvers, MA, USA)	9661
H3K27Ac	Cell Signaling Technology	8173
H3K27me3	Cell Signaling Technology	9733
H3K4me1	Cell Signaling Technology	5326
H3K4me2	Cell Signaling Technology	9725
H3K4me3	Cell Signaling Technology	9751
Histone H3	Cell Signaling Technology	4499
Ki67	Abcam (Cambridge, UK)	ab16667
p-Histone H3 (Ser28)	Cell Signaling Technology	9701

## Data Availability

Scripts are available upon request. All RNA-Seq data are available under GEO accession number [GSE320019].

## Supplementary Materials

The following supporting information can be downloaded at https://www.mdpi.com/article/10.3390/cells15080673/s1. Figure S1: Entinostat dose–response curves for OV207 and EFO21 monolayer cells treated for 7 days; Figure S2: 105C cell line spheroid reattachment after treatment with Panobinostat and Belinostat; Figure S3: Assessment of 105C spheroid reattachment upon treatment with Entinostat; Figure S4: Cell counts for 105C spheroids treated with 1 uM Entinostat for 3 days; Figure S5. Apoptosis in 105C cell line spheroids treated with Entinostat. Table S1: Entinostat EC50 values obtained for 14 human OCCC cell lines in monolayer culture.

## Abbreviations

The following abbreviations are used in this manuscript:

ACY	ACY-1215
ENT	Entinostat
EOC	Epithelial ovarian cancer
GSEA	Gene set enrichment analysis
HBSS	Hank’s balanced salt solution
HDAC	Histone deacetylase
HDACi	HDAC inhibitor
HDACis	HDAC inhibitors
HGSOC	High grade serous ovarian cancer
IC50	Half-maximal inhibitory concentration
Ki67	Antigen Kiel 67
ML	Monolayer
OCCC	Ovarian clear cell carcinoma
PCA	Principal component analysis
PVDF	Polyvinylidene difluoride
RIPA	Radioimmunoprecipitation assay
SPH	Spheroid
STR	Short tandem repeat
TBST	Tris-buffered saline with Tween 20
ULA	Ultralow attachment
VEH	Vehicle
