# Supplementary material for: HDAC Inhibition Induces Transient Phenotypic Inertia in Dormant OCCC Spheroids by Derepression of Cell Cycle Genes
Source: Cells. 2026 Apr 10;15(8):673. doi: 10.3390/cells15080673 (PMC13114744; doi:10.3390/cells15080673)
Supplement: Supplementary file 1 [file cells-15-00673-s001.zip › cells-4189384-supplementary.pdf]

Cheng et al. 2026; HDAC inhibition induces transient phenotypic inertia in dormant OCCC spheroids by derepression of cell cycle genes

S1

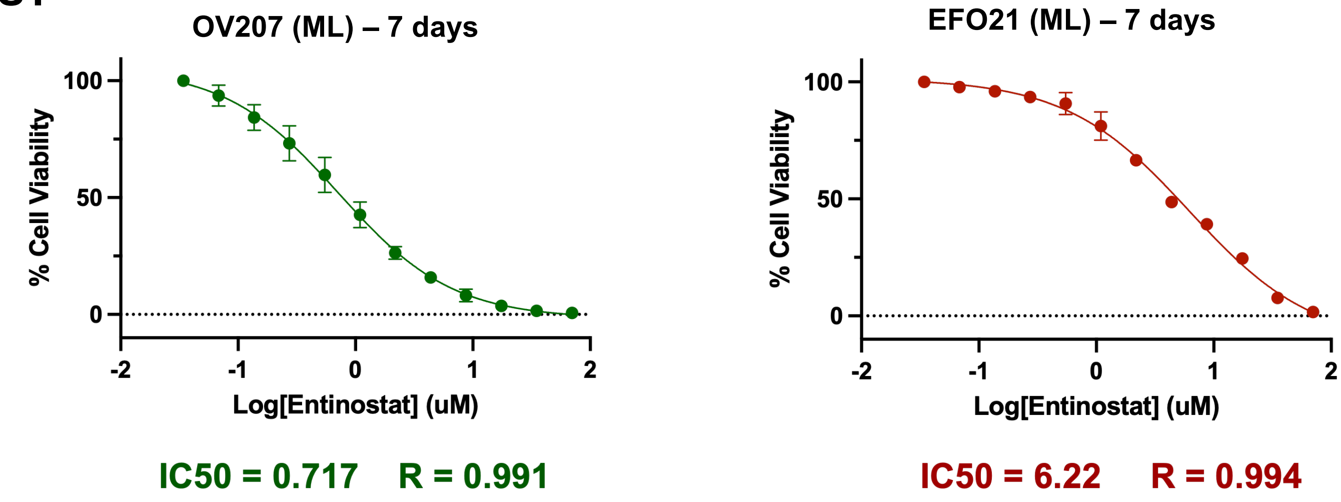

S2

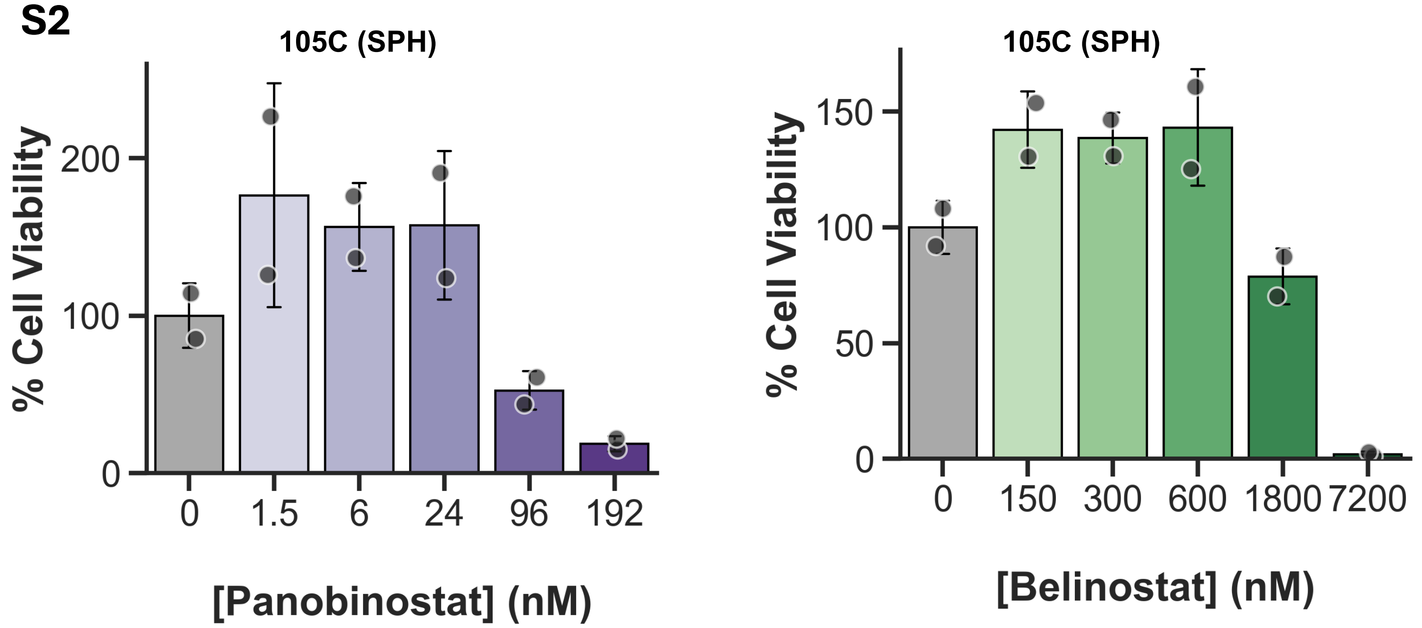

S3

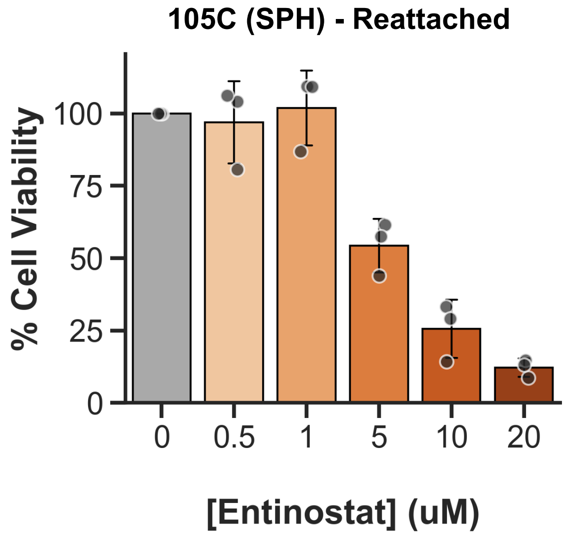

S4

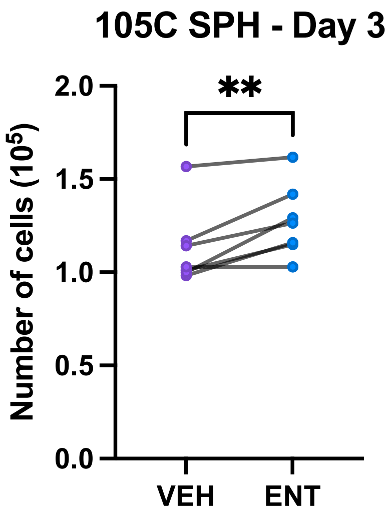

Cheng et al. 2026; HDAC inhibition induces transient phenotypic inertia in dormant OCCC spheroids by derepression of cell cycle genes

S5

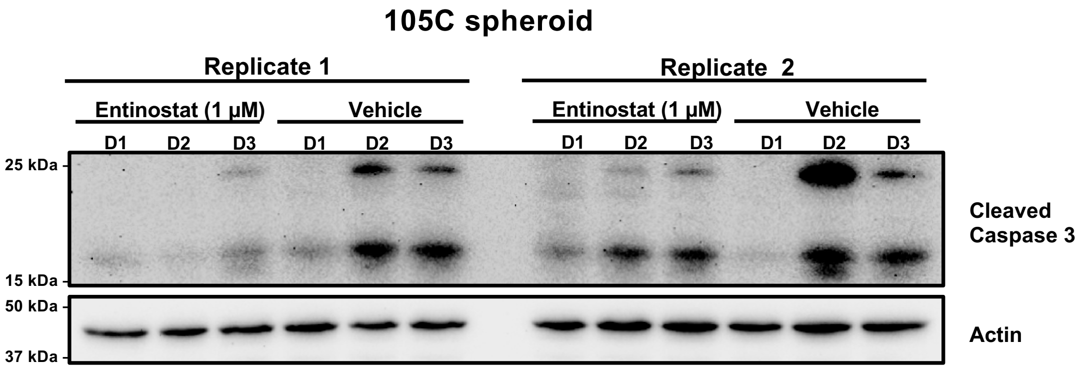

Figure S1. Entinostat dose-response curves for OV207 and EFO21 monolayer cells treated for 7 days. Cells were seeded at 1.5K cells/well in 96-well adherent culture and treated with increasing doses of Entinostat for 7 days. After treatment, AlamarBlue™ cell viability reagent was used to quantify treatment efficacy. GraphPad Prism was used to calculate IC50 concentrations.

Figure S2. 105C cell line spheroid reattachment after treatment with Panobinostat and Belinostat. 105C cells were seeded at 100K cells/well in 24-well ULA culture dishes to form spheroids and treated with increasing concentrations of Panobinostat or Belinostat at the time of seeding. After 3 days, all spheroid cells and media were transferred, well-for-well, into 24-well adherent culture plates for spheroid reattachment. Each well was also supplemented with 500 µL fresh media at this time. Spheroids were allowed to adhere to adherent culture plates over 24-48h. AlamarBlue™ cell viability reagent was used to quantify treatment response. At low doses of each HDAC inhibitor, 105C spheroid viability was enhanced. The highest doses of these agents caused a dramatic loss of cell viability. Points indicate technical replicates.

Figure S3. Assessment of 105C spheroid reattachment upon treatment with Entinostat.
105C cells were seeded at 100K cells per well in 24-well ULA dishes to form spheroids for 3 days. On day 3, spheroids were treated with Entinostat at the indicated doses for another 3 days, then the spheroids were transferred to adherent cell culture plates to allow spheroid attachment, well for well, into 24-well adherent culture plates. Two days after reattachment, cell viability was assessed with AlamarBlue™. One-way ANOVA, followed by Tukey's Multiple Comparisons Test was conducted on 3 independent experiments (n=3). Different letters (a, b, c) represent significantly different values. Bars show mean viability and SEM.

Figure S4. Cell counts for 105C spheroids treated with 1 uM Entinostat for 3 days.
Cell counts were pooled from two different experiments. For both experiments, 100K 105C cells were seeded per well in 24-well ULA to form spheroids and treated with 1 uM Entinostat or vehicle at the time of seeding. On day 3, trypan blue exclusion cell counting was performed. Paired cell counts are connected by a line. A paired t-test was performed, $p = 0.0031$.

Figure S5. Apoptosis in 105C cell line spheroids treated with Entinostat. Cells were seeded in 6-well ULA culture dishes at 500K cells/well and treated with 1 µM ENT at the time of seeding. Whole cell protein lysates were collected daily for 3 days and 20 µg/lane of whole cell lysate assessed by western blotting for cleaved caspase 3. Actin was used as a loading control.

OCCC Cell line	Entinostat IC50 (uM)
KOC-7c	1.92
ES2	3.19
12Z	0.83
TOV-21G	1.12
RMG-V	0.85
JHOC-5	2.68
RMG-I	1.99
OVTOKO	1.61
EFO27	1.62
105C	13.05
TU-OC-1	10.01
OVMANA	10.03
SMOV2	8.27
RMG-II	15.53

Table S1: Entinostat EC50 values obtained for 14 human OCCC cell lines in monolayer culture. These values pertain to Fig. 4, panel b bar graph.
